# Nav1.2 haplodeficiency in excitatory neurons causes absence-like seizures in mice

**DOI:** 10.1038/s42003-018-0099-2

**Published:** 2018-07-19

**Authors:** Ikuo Ogiwara, Hiroyuki Miyamoto, Tetsuya Tatsukawa, Tetsushi Yamagata, Tojo Nakayama, Nafiseh Atapour, Eriko Miura, Emi Mazaki, Sara J. Ernst, Dezhi Cao, Hideyuki Ohtani, Shigeyoshi Itohara, Yuchio Yanagawa, Mauricio Montal, Michisuke Yuzaki, Yushi Inoue, Takao K. Hensch, Jeffrey L. Noebels, Kazuhiro Yamakawa

**Affiliations:** 1 Laboratory for Neurogenetics, RIKEN Center for Brain Science, Wako, Saitama, 351-0198 Japan; 2 Department of Physiology, Nippon Medical School, Tokyo, 113-8602 Japan; 3 Laboratory for Neuronal Circuit Development, RIKEN Center for Brain Science, Wako, Saitama, 351-0198 Japan; 4 PRESTO, Japan Science and Technology Agency, Saitama, 332-0012 Japan; 5 Department of Pediatrics, Tohoku University School of Medicine, Sendai, 980-8574 Japan; 6 Department of Physiology, School of Medicine, Keio University, Tokyo, 160-8582 Japan; 7 Department of Neurology, Baylor College of Medicine, Houston, TX 77030 USA; 8 Department of Molecular and Human Genetics, Baylor College of Medicine, Houston, TX 77030 USA; 9 National Epilepsy Center, Shizuoka Institute of Epilepsy and Neurological Disorders, Shizuoka, 420-8688 Japan; 10 Laboratory for Behavioral Genetics, RIKEN Center for Brain Science, Wako, Saitama, 351-0198 Japan; 11 FIRST, Japan Science and Technology Agency, Saitama, 332-0012 Japan; 12 Department of Genetic and Behavioral Neuroscience, Gunma University Graduate School of Medicine, Maebashi, 371-8511 Japan; 13 CREST, Japan Science and Technology Agency, Saitama, 332-0012 Japan; 14 Section of Neurobiology, Division of Biological Sciences, University of California San Diego, 9500 Gilman Drive, La Jolla, CA 92093 USA; 15 Department of Molecular and Cellular Biology and Center for Brain Science, Harvard University, Cambridge, MA 02138 USA; 16 Department of Neurology, FM Kirby Neurobiology Center, Boston Children’s Hospital, Harvard Medical School, Boston, MA 02115 USA; 17 Department of Neuroscience, Baylor College of Medicine, Houston, TX 77030 USA; 18 Present Address: Division of Genetics and Genomics, Boston Children’s Hospital, Harvard Medical School, Boston, MA 02115 USA; 19 Present Address: Department of Medicine (Royal Melbourne Hospital), Melbourne Brain Centre, University of Melbourne, Parkville, VIC 3050 Australia; 20 Present Address: Neurology Department, Shenzhen Children’s Hospital, 518026 Guangdong, China

## Abstract

Mutations in the *SCN2A* gene encoding a voltage-gated sodium channel Nav1.2 are associated with epilepsies, intellectual disability, and autism. *SCN2A* gain-of-function mutations cause early-onset severe epilepsies, while loss-of-function mutations cause autism with milder and/or later-onset epilepsies. Here we show that both heterozygous *Scn2a*-knockout and knock-in mice harboring a patient-derived nonsense mutation exhibit ethosuximide-sensitive absence-like seizures associated with spike-and-wave discharges at adult stages. Unexpectedly, identical seizures are reproduced and even more prominent in mice with heterozygous *Scn2a* deletion specifically in dorsal-telencephalic (e.g., neocortical and hippocampal) excitatory neurons, but are undetected in mice with selective *Scn2a* deletion in inhibitory neurons. In adult cerebral cortex of wild-type mice, most Nav1.2 is expressed in excitatory neurons with a steady increase and redistribution from proximal (i.e., axon initial segments) to distal axons. These results indicate a pivotal role of Nav1.2 haplodeficiency in excitatory neurons in epilepsies of patients with *SCN2A* loss-of-function mutations.

## Abstract (web summary)

Ikuo Ogiwara et al. find that nonsense mutations in the gene coding for the voltage-gated sodium channel Nav1.2, *SCN2A*, cause absence-like seizures in mice. They also find that Nav1.2 haplodeficiency in excitatory, but not in inhibitory, neurons contributes to epileptogenesis.

## Introduction

Voltage-gated sodium channels in neurons play essential roles in the generation and propagation of action potentials. These channels consist of one pore-forming α subunit and one or two accessory β subunits. In the mammalian brain, four α subunits, namely, Nav1.1, 1.2, 1.3, and 1.6 encoded by *SCN1A*, *2A*, *3A*, and *8A*, respectively, are expressed at high levels.

Mutations in voltage-gated sodium channel genes have been described in patients with a wide spectrum of neurological disorders including epilepsy. The first mutation in *SCN2A* was discovered in a patient with atypical generalized epilepsy with febrile seizures plus^1^. Subsequently, inherited *SCN2A* mutations were found in families with benign familial neonatal-infantile seizures^2,3^. We further reported a nonsense mutation *SCN2A*-R102* (RX) in a patient with epileptic encephalopathy, autism spectrum disorder (ASD) and intellectual disability^4^, which was the first report of a de novo *SCN2A* mutation in a patient with ASD or intellectual disability. Subsequently, we and others reported a number of de novo *SCN2A* mutations in patients with neurological disorders such as epileptic encephalopathy including Ohtahara syndrome, West syndrome, Lennox Gastaut syndrome^5–9^, ASD^10,11^, intellectual disability^12,13^, and schizophrenia^14,15^. Recent large-scale whole exome sequencing studies further revealed that *SCN2A* is the gene showing the most frequent and common de novo mutations among these patients^16–20^.

Although mutations of *SCN1A* have also been described in patients with epileptic encephalopathy, intellectual disability, and ASD^8,9,18,21,22^, the distributions of Nav1.1 and Nav1.2 are highly distinct from each other in brain. In neocortex, hippocampus and cerebellum, Nav1.1 is dominantly expressed in parvalbumin-positive GABAergic neurons such as fast-spiking (FS) basket cells and Purkinje cells in their axonal features^23–25^, while Nav1.2 is robustly expressed in glutamatergic neurons including most neocortical pyramidal cells at their axon initial segments^26–29^. Nav1.2 is densely expressed in unmyelinated axons of neurons in hippocampal dentate and cerebellar granule cells^30,31^, although in striatum Nav1.2 is present at unmyelinated axons of GABAergic medium spiny neurons^32^. We recently reported that Nav1.1 and Nav1.2 are expressed in a mutually exclusive manner not only in neocortex, hippocampus, and cerebellum, but also in striatum, where medium spiny neurons are Nav1.2-positive and presumed FS inhibitory interneurons are Nav1.1-positive^29^. In globus pallidus, all GABAergic neurons are Nav1.1-positive and the dense Nav1.2 signals are derived from axonal fibers of striatal medium spiny neurons^29^. Li and colleagues^33^ reported that Nav1.2 is expressed in neocortical somatostatin-positive inhibitory neurons but not in parvalbumin-positive neurons. However, we found that Nav1.2 is expressed in caudal ganglionic eminence-derived vasoactive intestinal peptide-positive or reelin-positive/somatostatin-negative inhibitory neurons in neocortex and hippocampus, but is not expressed in parvalbumin or somatostatin-positive neurons, which are medial ganglionic eminence-derived inhibitory neurons^29^.

Contrary to *SCN1A* loss-of-function mutations in patients with severe epilepsies such as Dravet syndrome^22,34^, *SCN2A* gain-of-function (increased or accelerated, but not toxic) has recently been recognized as a cause of early infantile-onset severe epileptic encephalopathies such as Ohtahara syndrome, whereas loss-of-function *SCN2A* mutations underlie ASD or intellectual disability with later-onset mild epilepsy or without epilepsy^22,35,36^. Given that the predominant expression of Nav1.1 is in inhibitory neurons and that of Nav1.2 is in excitatory neocortical/hippocampal neurons, it seems reasonable that *SCN1A* loss- or *SCN2A* gain-of-function mutations lead to epilepsies. However, it still remains unclear why *SCN2A* loss-of-function mutations also cause epilepsies.

A mouse transgenic line *Scn2*a^Q54^ exhibiting partial seizures originating in hippocampus^37^ has long been used as a model for diseases caused by *SCN2A* mutations. This mouse model harbors a GAL879-881QQQ *Scn2a* gain-of-function mutation and the mutant protein is ectopically expressed under the control of the rat promoter for a neuron-specific enolase gene, while intrinsic *Scn2a* genes remain intact. In mice with genuine *Scn2a* deficiency, no epileptic seizures have been described so far^38^.

In this study, we discovered that *Scn2a* haploinsufficient mice show a mild spontaneous epileptic phenotype of absence-like seizures. Contrary to the previous proposal that loss-of-function *Scn2a* mutations may reduce excitability of Nav1.2 expressing inhibitory neurons and thereby lead to epileptic seizures^33^, we show here that the epileptic phenotypes in mice with *Scn2a* deficiency depend on Nav1.2 deficiency in excitatory neurons, suggesting critical contributions of impaired functions of excitatory neurons to the pathophysiology of epileptic seizures associated with *SCN2A* mutations.

## Results

### Absence-like seizures in Nav1.2 haploinsufficient mice

**Fig. 1:**
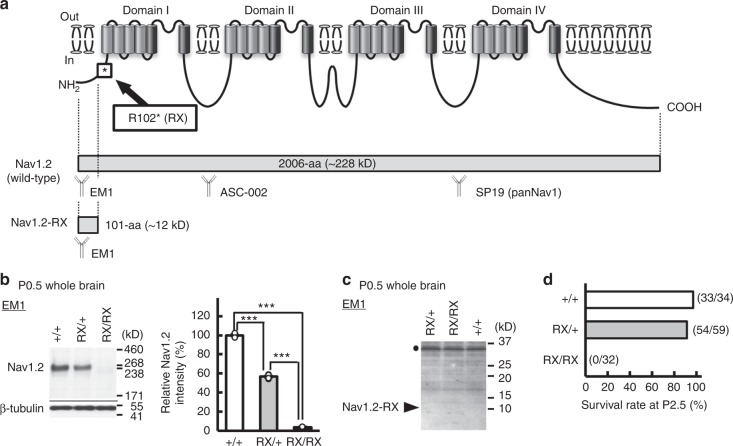
A pathogenic *Scn2a* nonsense mutation inactivated the mutated allele. **a** Schematic of the voltage-gated sodium channel Nav1.2, showing the location of R102* (RX) nonsense mutation. Full-length wild type Nav1.2 is composed of 2006-amino acid (aa) residues with the predicted molecular weight of ~228 kD. The RX mutation can cause a truncated peptides, Nav1.2-RX, consisting of the first 101-aa residues of Nav1.2 with the theoretical molecular weight of ~12 kD. Epitope locations for the anti-Nav1.2 (EM-1, ASC-002) and anti-pan Nav1 (SP19) antibodies are indicated. **b**, **c** The RX allele was effectively inactivated in *Scn2a* knock-in mice. Western blot analyses of P0.5 whole brain membrane (**b**) and cytosolic (**c**) fractions were performed using EM-1. **b** Full-length Nav1.2 was moderate and negligible in *Scn2a*^RX/+^ (RX/+, *N* = 4) and *Scn2a*^RX/RX^ (RX/RX, *N* = 3) mice, respectively, compared with that in *Scn2a*^+/+^ mice (+/+, *N* = 3) [one-way analysis of variance; genotype: F (2, 7) = 1737, ***P = 0.00000000036, Tukey’s test; *Scn2a*^+/+^ *vs*. *Scn2a*^RX/+^ ***P < 0.0001: *Scn2a*^+/+^ *vs*. *Scn2a*^RX/RX^, ***P < 0.0001: *Scn2a*^RX/+^ *vs*. *Scn2a*^RX/RX^, ***P < 0.0001]. Full-length Nav1.2 ran slower than its predicated molecular weight. Mean Nav1.2 signal intensities are represented as percentages relative to that of *Scn2a*^+/+^ littermates (100%). β-tubulin was used as internal control. **c** No bands appeared at expected size of ~12 kD (arrowhead) for Nav1.2-RX in any genotypes. The bands at ~36 kD (black dot) were non-specific. **d** Survival rates at P2.5 of *Scn2a*^+/+^ (*N* = 34), *Scn2a*^RX/+^ (*N* = 59) and *Scn2a*^RX/RX^ mice (*N* = 32). All RX/RX mice died before P2.5. Data represent means ± SEM, ***P < 0.001

We have previously suggested that a truncated non-functional peptide (Nav1.2-RX) consisting of the N-terminal 101 amino acid residues might cause dominant negative Nav1.2 suppression leading to intractable seizures in a patient with the *Scn2a*-RX mutation^4^. In order to test this hypothesis, we generated knock-in mice carrying the RX mutation (Fig. 1a and Supplementary Fig. 1a-c) and compared their phenotypes with those of previously reported *Scn2a*-knockout (KO) mice carrying the disrupted exon 1 with an insertion of neo cassette^38^. Western blot analyses of whole brain lysate at postnatal day (P) 0.5, using a newly-generated anti-N-terminal-Nav1.2 antibody (EM1) and an anti-internal (ASC-002) Nav1.2 antibody showed that wild-type Nav1.2 was highly expressed in wild-type (*Scn2a*^+/+^) mice, moderately expressed in heterozygote (*Scn2a*^RX/+^) mice and negligibly expressed in homozygote (*Scn2a*^RX/RX^) mice (Fig. 1b and Supplementary Fig. 1d). Meanwhile, truncated Nav1.2-RX was undetected in *Scn2a*^RX/+^ and *Scn2a*^RX/RX^ mice (Fig. 1c), suggesting that the mutated *Scn2a* allele was inactivated, presumably by nonsense-mediated mRNA decay. Western blot analyses using the anti-pan Nav1 antibody (SP19) also showed reduced expression levels of Nav1 (total voltage-gated sodium channel alpha-subunits) in *Scn2a*^RX/RX^ mice, compared with *Scn2a*^+/+^ mice (Supplementary Fig. 1e), consistent with inactivation of the *Scn2a* mutated allele. Like homozygous *Scn2a* KO (*Scn2a*^KO/KO^) mice^38^, *Scn2a*^RX/RX^ pups were born in the approximately expected Mendelian ratios, but all died within two days after birth (Fig. 1d). *Scn2a*^RX/+^ mice were viable and fertile, and had normal life spans, as observed in *Scn2a*^KO/+^ mice^38^.

**Fig. 2:**
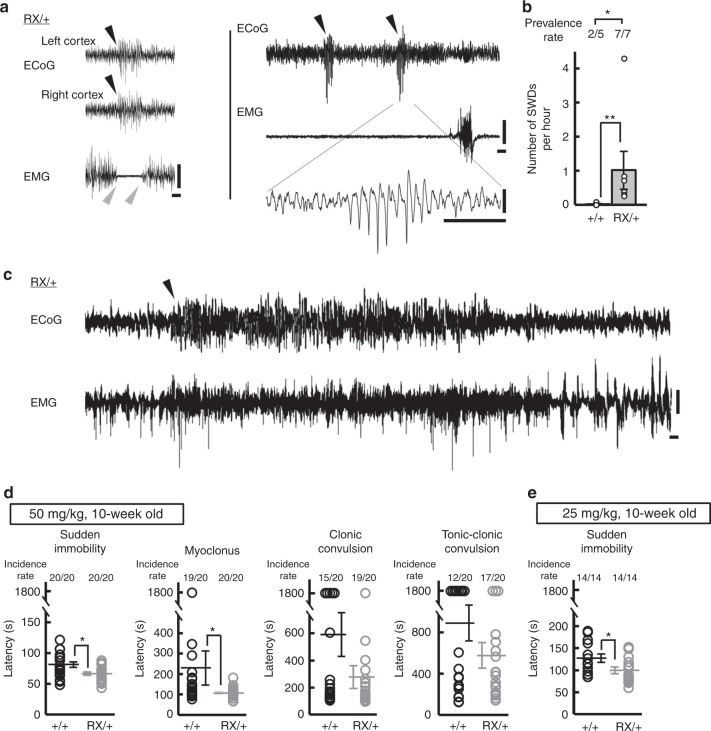
The pathogenic *Scn2a* nonsense mutation caused absence-like seizures with SWDs in mice. **a** Representative traces of somatosensory ECoG/EMG recordings in 6–11 weeks-old *Scn2a*^RX/+^ mice (*N* = 7). Behavioral arrest during waking state associated with ECoG epileptiform SWDs (left). Black arrowheads indicate the onset of SWD. Gray arrowheads indicate the onset and end of EMG suppression. Positivity was plotted up. **b** Frequencies of SWDs during a 24 h ECoG recording period in *Scn2a*^+/+^ (*N* = 5) and *Scn2a*^RX/+^ (*N* = 7) mice. **c** An episode of non-convulsive epileptiform discharges detected in 1 out of 7 *Scn2a*^RX/+^ mice. An arrowhead indicates the onset of epileptiform discharge. Positivity was plotted up. **d**, **e** Seizure susceptibility to PTZ in *Scn2a*^RX/+^ and *Scn2a*^+/+^ mice (10 weeks of age). The latencies to the first appearance of absence seizure-like sudden immobility, myoclonus, clonic convulsion and tonic-clonic convulsion after intraperitoneal administration of PTZ at dose of 50 (**d**, *N* = 20, each genotype) and 25 (**e**, *N* = 14, each genotype) mg per kg body weight. *Scn2a*^RX/+^ mice had significantly shorter latencies to absence-like sudden immobility (Mann-Whitney test, 50 mg per kg, sudden immobility, U = 106, *P = 0.0100, myoclonus, U = 116.5, *P = 0.0231, clonic convulsion, U = 162, *P* = 0.3098, tonic-clonic convulsion, U = 175, *P* = 0.5023; 25 mg per kg, sudden immobility, U = 53, *P = 0.0383). Data represent means ± SEM, *P < 0.05, **P < 0.01. Scale bars: **a**, **c** vertical 0.5 mV; horizontal 1 s

Although visual inspection did not detect behavioral seizures in *Scn2a*^RX/+^ mice, long-term electrocorticography (ECoG)-electromyography (EMG) recordings at 6–11 weeks of age revealed frequent abnormal ECoG patterns, typically <1 s bursts of high-amplitude bilateral spike-and-wave discharges (SWDs) associated with EMG suppression indicating behavioral arrest (Fig. 2a). These features of *Scn2a*^RX/+^ mice closely resembled epileptiform discharges observed in rodent models of absence epilepsy, except that the duration of SWD episodes in *Scn2a*^RX/+^ mice (mean ± SEM, 0.71 ± 0.04 sec, 70 ECoG discharges) was much shorter than those (usually more than 2 s) in other rodent models^39–41^. 24-h ECoG-EMG recordings showed that *Scn2a*^RX/+^ mice had a higher prevalence and a greater incidence of epileptiform discharges than *Scn2a*^+/+^ mice (prevalence rate: Fisher’s exact test, *P = 0.0455, hourly incidence: Mann–Whitney test, *U* = 0, **P = 0.0025, Fig. 2b). ECoG–EMG recordings also detected one prolonged non-convulsive seizure with duration of 50 s in 1 out of 7 *Scn2a*^RX/+^ mice examined (Fig. 2c). Analysis of susceptibility to induced seizures by a chemoconvulsant, a GABA_A_ receptor antagonist pentylenetetrazol (PTZ, 50 mg per kg or 25 mg per kg), revealed a shorter latency to the appearance of absence seizure-like sudden immobility in *Scn2a*^RX/+^ than in *Scn2a*^+/+^ mice (Fig. 2d, e).

**Fig. 3:**
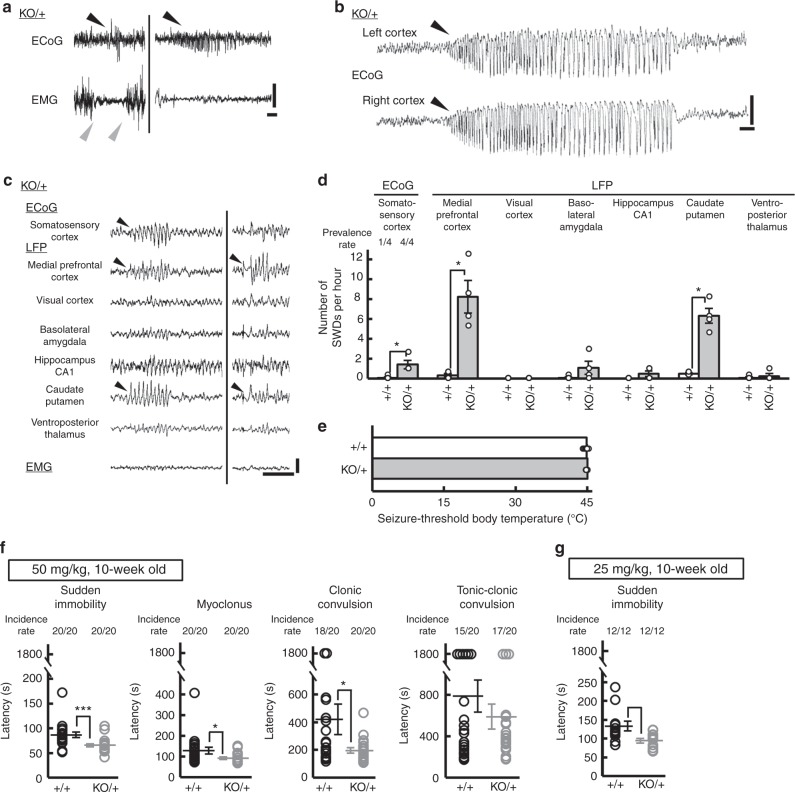
Heterozygous *Scn2a* knockout mice showed absence-like seizures with SWDs. **a** Representative traces of ECoG/EMG recordings from 10–27 weeks-old *Scn2a*^KO/+^ (KO/+) mice (*N* = 6). Black arrowheads indicate the onset of SWD. Gray arrowheads indicate the onset and end of EMG suppression. **b** A representative trace of prolonged non-convulsive seizure. ECoG recordings detected 2 episodes of prolonged non-convulsive seizure with duration of 30–45 s in 2 out of 6 *Scn2a*^KO/+^ mice, which were neither accompanied by convulsions, nor followed by post-ictal depression. **c** Representative ECoG/EMG/LFP recordings during an SWD episode in *Scn2a*^KO/+^ mice. Epileptiform discharges with large amplitudes are seen in mPFC and CPu. Positivity was plotted up (**a**, **b**, **c**). **d** Quantification of ECoG SWDs and LFP epileptiform discharges [3-hour recording, light period, *Scn2a*^+/+^, *Scn2a*^KO/+^ (*N* = 4, each genotype)]. Mann–Whitney test, medial prefrontal cortex: *U* = 0, *P = 0.0286; visual cortex: *U* = 8, *P* > 0.9999; basolateral amygdala: *U* = 3, *P* = 0.2571; hippocampus CA1: *U* = 4, *P* = 0.4286; caudate putamen: *U* = 0, *P = 0.0286; ventroposterior thalamus: *U* = 7.5, *P* > 0.9999. **e** Thresholds of body temperature for hyperthermia-induced seizures did not differ between *Scn2a*^KO/+^ and *Scn2a*^+/+^ mice (4-week-old, *N* = 10, each genotype) [unpaired *t*-test, *t*(18) = 1.149, *P* = 0.2658]. **f**, **g** Increased seizure susceptibility to PTZ in 10-week-old *Scn2a*^KO/+^ mice. Latencies to the first appearance of sudden immobility, myoclonus, clonic convulsion, and tonic-clonic convulsion after administrating of PTZ at doses of 50 (**f**, *N* = 20, each genotype) or 25 (**g**, *N* = 12, each genotype) mg per kg body weight. The latencies to the appearance of sudden immobility, myoclonus and clonic convulsion were shorter in *Scn2a*^KO/+^ than in *Scn2a*^+/+^ mice (Mann-Whitney test, 50 mg per kg, sudden immobility, *U* = 69.5, ***P = 0.0002, myoclonus, *U* = 110.5, *P = 0.0145, clonic convulsion, *U* = 119, *P = 0.0278, tonic-clonic convulsion, *U* = 188.5, *P* = 0.7624; 25 mg per kg, sudden immobility, *U* = 26.5, **P = 0.0071). Data represent means ± SEM, *P < 0.05, **P < 0.01, ***P < 0.001. Scale bars: (**a**–**c**) vertical 0.5 mV; horizontal 1 s

**Fig. 4:**
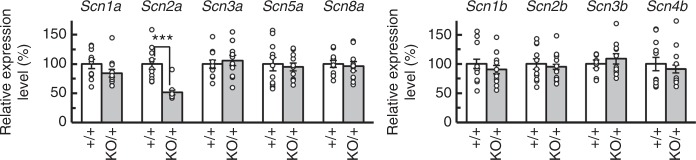
*Scn2a* haplodeficiency did not alter expression levels of other sodium channel subunits. Quantitative RT-PCR analyses of brain mRNAs prepared from P14.5 *Scn2a*^+/+^ and *Scn2a*^KO/+^ mice (*N* = 11, each genotype). *Scn2a* mRNA expression in *Scn2a*^KO/+^ whole brain was reduced to about 50% level of that in *Scn2a*^+/+^ mice while there were no significant changes in *Scn1a*, *Scn3a*, *Scn5a*, *Scn8a*, *Scn1b*, *Scn2b*, *Scn3b*, and *Scn4b* mRNA expression levels in *Scn2a*^KO/+^, compared with those in *Scn2a*^+/+^ mice [unpaired t-test, *Scn1a*; *t*(20) = 1.461, *P* = 0.1595, *Scn2a*; *t*(20) = 5.250, ***P = 0.000039, *Scn3a*; *t*(20) = 0.5066, *P* = 0.6180, *Scn5a*; *t*(20) = 0.4223, *P* = 0.6773, *Scn8a*; *t*(20) = 0.3952, *P* = 0.6969, *Scn1b*; *t*(20) = 0.7407, *P* = 0.4675, *Scn2b*; *t*(20) = 0.4259, *P* = 0.6747, *Scn3b*; *t*(20) = 0.8664, *P* = 0.3965, *Scn4b*; *t*(20) = 0.5273, *P* = 0.6038]. White and gray bars represent *Scn2a*^+/+^ and *Scn2a*^KO/+^ mice, respectively. Data represent means ± SEM, ***P < 0.001

We next investigated whether *Scn2a*^KO/+^ mice also have absence-like seizures. While visual inspections did not reveal any discernible behavioral seizures in *Scn2a*^KO/+^ mice, consistent with a previous study^38^, ECoG-EMG recordings from *Scn2a*^KO/+^ mice at 10–27 weeks of age detected absence-like seizures with SWDs associated with EMG suppression (Fig. 3a) as observed in *Scn2a*^RX/+^ mice. ECoG monitoring further revealed two prolonged non-convulsive seizures with duration of 30–45 s in 2 out of 6 *Scn2a*^KO/+^ mice examined (Fig. 3b). 3-h ECoG-multisite local field potential (LFP) recordings showed that *Scn2a*^KO/+^ mice had a greater incidence of ECoG SWDs than *Scn2a*^+/+^ mice (ECoG on somatosensory cortex, prevalence rate: Fisher’s exact test, *P* = 0.1492, hourly incidence: Mann–Whitney test, *U* = 0, *P = 0.0286) (Fig. 3c, d). Moreover, ECoG-multisite LFP recordings revealed the predominant appearance of LFP epileptiform discharges in medial prefrontal cortex (mPFC) and caudate putamen (CPu) (Fig. 3c, d). While the incidence and duration of epileptiform discharges did not significantly differ between *Scn2a*^RX/+^ and *Scn2a*^KO/+^ mice (hourly incidence: *Scn2a*^RX/+^, 1.018 ± 0.552; *Scn2a*^KO/+^, 1.417 ± 0.417; Mann–Whitney test, *U* = 4, *P* = 0.0606, 95.76% confidence interval [−2.292, 3.292], duration: *Scn2a*^RX/+^, 0.707 ± 0.044 sec; *Scn2a*^KO/+^, 0.578 ± 0.046 sec; Mann–Whitney test, *U* = 1134, *P* = 0.0982, 95.04% confidence interval [−0.016, 0.226], *Scn2a*^RX/+^, *N* = 7, 70 SWD episodes; *Scn2a*^KO/+^, *N* = 4, 40 SWD episodes), the maximum amplitude and spike numbers during a SWD episode were larger in *Scn2a*^RX/+^ mice than in *Scn2a*^KO/+^ mice (maximum amplitude: *Scn2a*^RX/+^, 0.568 ± 0.019 mV; *Scn2a*^KO/+^, 0.453 ± 0.014 mV; Mann–Whitney test, *U* = 736.5, ***P < 0.0001, 95.04% confidence interval [−0.053, 0.142], number of spikes: *Scn2a*^RX/+^, 5.33 ± 0.27; *Scn2a*^KO/+^, 4.20 ± 0.21; Mann–Whitney test, *U* = 968, *P* = 0.0057, 95.04% confidence interval [0.00, 1.00], *Scn2a*^RX/+^, *N* = 7, 70 SWD episodes; *Scn2a*^KO/+^, *N* = 4, 40 SWD episodes). Given that incidence and duration of SWD episodes were 1.018 ± 0.552 episodes per hour and 0.71 ± 0.04 sec in *Scn2a*^RX/+^ mice and 1.417 ± 0.417 episodes per hour and 0.58 ± 0.05 sec in *Scn2a*^KO/+^ mice, the percentage of total recording period displaying seizures was calculated to be around 0.02% for both lines. The susceptibility to hyperthermia-induced seizure was not altered in *Scn2a*^KO/+^ compared to *Scn2a*^+/+^ mice (Fig. 3e). Analysis of susceptibility to PTZ (50 mg per kg or 25 mg per kg) induced seizures showed a shorter latency to the appearance of absence seizure-like sudden immobility, myoclonus, and clonic convulsion in *Scn2a*^KO/+^ than in *Scn2a*^+/+^ mice (Fig. 3f, g). Nav1.2 haploinsufficiency in *Scn2a*^KO/+^ did not alter the basal expression levels of other sodium channel subunits (Fig. 4). Taken together, these results suggest that Nav1.2 haploinsufficiency is the pathological basis for the absence-like seizures in these mice.

### Seizures in mice with Nav1.2 deletion in excitatory neurons

Nav1.2 is expressed in caudal ganglionic eminence-derived inhibitory neurons, such as vasoactive intestinal peptide-positive or reelin-positive/somatostatin-negative inhibitory neurons^29^, and in pyramidal neurons^26–29^. In order to evaluate the relative impact of Nav1.2 haploinsufficiency in excitatory and inhibitory neurons on the absence-like seizures observed in *Scn2a*-knock-in and KO heterozygous mice, we examined a series of *Scn2a* conditional KO mice. At first, we generated a mouse line with a floxed *Scn2a* allele containing two loxP cassettes placed on either side of coding exon 2 (Supplementary Fig. 2a-c). Mice homozygous for the floxed allele (*Scn2a*^fl/fl^) were viable, showed no obvious abnormal phenotypes, and expressed normal levels of Nav1.2 (Supplementary Fig. 2d). Next, we generated a constitutively deleted *Scn2a* allele by crossing *Scn2a*^fl/fl^ with EIIa-Cre line, in which the Cre-loxP recombination occurs in germline cells^42^. Nav1.2 expression levels in whole brain were high, moderate and undetectable in wild-type (*Scn2a*^+/+^), heterozygous (*Scn2a*^del/+^) and homozygous mice (*Scn2a*^del/del^), respectively (Supplementary Fig. 2e). *Scn2a*^del/+^ was viable and fertile, while *Scn2a*^del/del^ died within two days after birth (Supplementary Fig. 2f), similarly to those of *Scn2a*-knock-in and -KO mice.

**Fig. 5:**
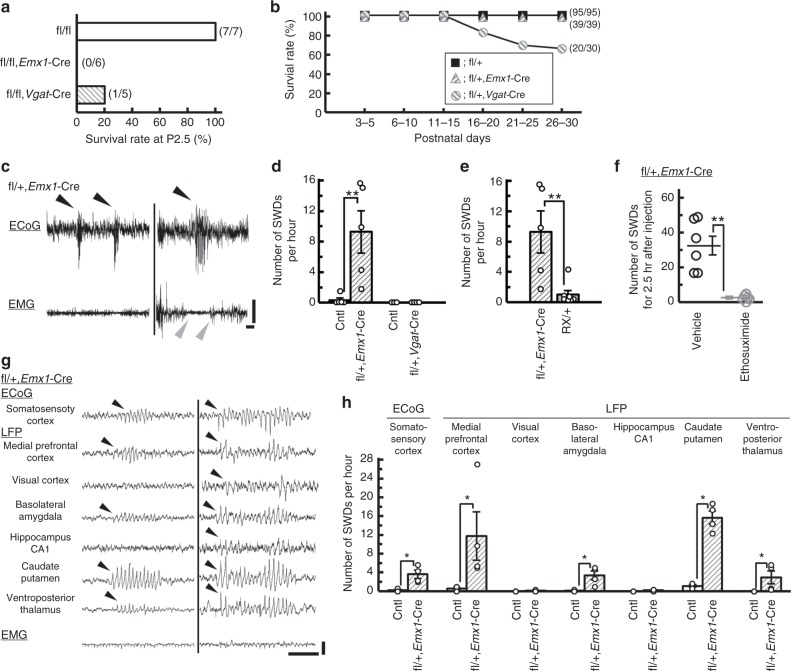
*Scn2a* deletion in dorsal telencephalic excitatory but not global inhibitory neurons triggered SWDs in mice. **a** Survival rates at P2.5 of *Scn2a*^fl/fl^ (*N* = 7), *Scn2a*^fl/fl^/*Emx1*-Cre (*N* = 6) and *Scn2a*^fl/fl^/*Vgat*-Cre mice (*N* = 5). All *Scn2a*^fl/fl^/*Emx1*-Cre and all but one *Scn2a*^fl/fl^/*Vgat*-Cre mice died before P2.5. One *Scn2a*^fl/fl^/*Vgat*-Cre survivor died at P8.5. **b** Survival curves during P3–30 of *Scn2a*^fl/+^/*Emx1*-Cre (*N* = 39), *Scn2a*^fl/+^/*Vgat*-Cre (*N* = 30) and *Scn2a*^fl/+^ mice (*N* = 95). About 30% of *Scn2a*^fl/+^/*Vgat*-Cre mice suffered premature death between P16 and P25. **c** Representative ECoG/EMG traces in *Scn2a*^fl/+^/*Emx1*-Cre mice. SWDs during waking were often associated with behavioral arrest (right). Black arrowheads indicate the onsets of SWDs. Gray arrowheads indicate the onset and end of behavioral arrest. **d**, **e** Frequencies of SWDs during 24 h ECoG recordings in *Scn2a*^fl/+^/*Emx1*-Cre (*N* = 5) and littermate controls (Cntl) (2 *Scn2a*^fl/+^, 3 *Scn2a*^+/+^/*Emx1*-Cre, *N* = 5), *Scn2a*^fl/+^/*Vgat*-Cre (*N* = 4) and littermate Cntl (2 *Scn2a*^fl/+^, 1 *Scn2a*^+/+^/*Vgat*-Cre, *N* = 3), and *Scn2a*^RX/+^ mice (*N* = 7). All recorded mice were over 8 weeks of age. **f** Ethosuximide (33.3 mg mL^−1^ in saline, 200 mg per kg, *i.p*.) efficiently suppressed SWDs in *Scn2a*^fl/+^/*Emx1*-Cre mice (*N* = 6). **g**, **h** LFP recordings from 3–6-month-old *Scn2a*^fl/+^/*Emx1*-Cre (*N* = 4) and littermate Cntl (2 *Scn2a*^+/+^, 2 *Scn2a*^fl/+^, 3 *Scn2a*^+/+^/*Emx1*-Cre, *N* = 7). **g** Representative ECoG/EMG/LFPs traces in *Scn2a*^fl/+^/*Emx1*-Cre mice. **h** Epileptiform discharges were predominantly detected in medial prefrontal cortex and caudate putamen of *Scn2a*^fl/+^/*Emx1*-Cre mice (Mann–Whitney test, ECoG, somatosensory cortex: *U* = 0, *P = 0.0286; LFP, medial prefrontal cortex: *U* = 0, *P = 0.0286; visual cortex: *U* = 6, *P* > 0.999; basolateral amygdala: *U* = 0, *P = 0.0268; hippocampus CA1: *U* = 4, *P* = 0.4286; caudate putamen: *U* = 0, *P = 0.0268; ventroposterior thalamus: *U* = 0, *P = 0.0286). Data represent means ± SEM, *P < 0.05, **P < 0.01. Scale bars: (**c**, **g**) vertical 0.5 mV; horizontal 1 s

We then crossed *Scn2a*^fl/fl^ with an *Emx1*-Cre driver line^43^, in which Cre recombinase is expressed in excitatory neurons of dorsal telencephalon consisting of neocortex, hippocampus, amygdala, piriform cortex, entorhinal cortex and olfactory bulb, and Cre-mediated recombination is detectable at embryonic day 10. We also deleted *Scn2a* gene selectively in global inhibitory neurons by crossing *Scn2a*^fl/fl^ mice with a *Vgat*-Cre driver line^25^. PCR analyses of DNAs from *Scn2a*^fl/fl^/*Emx1*-Cre and *Scn2a*^fl/fl^/*Vgat*-Cre whole brains at P0.5 verified Cre-dependent recombination of the floxed *Scn2a* allele (Supplementary Fig. 3a). Western blot analyses of Nav1.2 expression in P0.5 whole brain showed a ~ 30% reduction in *Scn2a*^fl/fl^/*Emx1*-Cre and a ~ 60% reduction in *Scn2a*^fl/fl^/*Vgat*-Cre, compared with *Scn2a*^fl/fl^ mice (Supplementary Fig. 3b, c), suggesting that *Emx1*-Cre or *Vgat*-Cre-mediated recombination effectively occurs in the perinatal period. Immunohistochemistry showed that Nav1.2-immunoreactive fibers and puncta scattered in cortical plate and hippocampal stratum pyramidale and radiatum, presumably corresponding to axon initial segments (AISs) of excitatory neurons^26–29^, were strongly detected in P0.5 *Scn2a*^fl/fl^ controls, but virtually undetectable in P0.5 *Scn2a*^fl/fl^/*Emx1*-Cre mice (Supplementary Fig. 3d-i). Immunohistochemistry furthermore showed that Nav1.2-immunoreactive fibers in neocortical layer I and hippocampal stratum lacunosum-moleculare, which putatively correspond to AISs of reelin-positive/somatostatin-negative inhibitory neurons^29^, were clearly observed in P9.5 *Scn2a*^fl/+^ controls, but almost absent in P9.5 *Scn2a*^fl/fl^/*Vgat*-Cre mice (Supplementary Fig. 3j-o). Like homozygous *Scn2a*-deficient mice, *Scn2a*^fl/fl^/*Emx1*-Cre and *Scn2a*^fl/fl^/*Vgat*-Cre mice were born, while all (*n* = 6) *Scn2a*^fl/fl^/*Emx1*-Cre and most (4 out of 5) *Scn2a*^fl/fl^/*Vgat*-Cre mice died within two days after birth (Fig. 5a). *Scn2a*^fl/+^/*Emx1*-Cre mice were viable and fertile and showed no obvious phenotypic abnormalities, while *Scn2a*^fl+^/*Vgat*-Cre mice were viable but approximately one-third (9 out of 30) suffered sudden death for unknown reasons between P18 and 25 (Fig. 5b).

ECoG-EMG recordings at 6–8 weeks of age detected absence-like seizures with SWDs with EMG suppression in *Scn2a*^fl/+^/*Emx1*-Cre, but not in *Scn2a*^fl/+^/*Vgat*-Cre mice (Mann–Whitney test, Cntl vs. *Scn2a*^fl/+^/*Emx1*-Cre: *U* = 0, **P = 0.0075) (Fig. 5c, d). Absence-like seizures appeared ~ 10 × more frequently in *Scn2a*^fl/+^/*Emx1*-Cre than in *Scn2a*^RX/+^ mice (Mann–Whitney test, *Scn2a*^RX/+^ vs. *Scn2a*^fl/+^/*Emx1*-Cre: U = 2, **P = 0.0088) (Fig. 5e). Absence-like seizures in *Scn2a*^fl/+^/*Emx1*-Cre mice were effectively suppressed by intraperitoneal injection of ethosuximide, a specific anti-absence drug (paired *t*-test, vehicle vs. ethosuximide, *t*(5) = 5.683, **P = 0.0024) (Fig. 5f). Simultaneous ECoG-multisite LFP recordings from *Scn2a*^fl/ +^/*Emx1*-Cre mice revealed pronounced appearance of LFP epileptiform discharges in mPFC and CPu (Fig. 5g, h), as observed in *Scn2a*^KO/+^ mice. Altogether, these observations indicate that Nav1.2 haplodeficiency in dorsal-telencephalic excitatory but not in global inhibitory neurons is sufficient to produce absence-like seizures.

### Redistribution of Nav1.2 during brain development

**Fig. 6:**
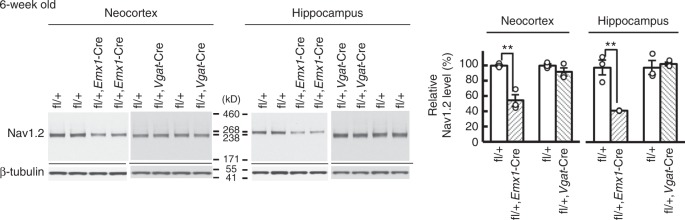
*Scn2a* haplodeficiency in dorsal telencephalic excitatory but in those in global inhibitory neurons reduced neocortical and hippocampal Nav1.2 expression levels. Western blot analyses of 6-weeks neocortex or hippocampus for *Scn2a*^fl/+^/*Emx1*-Cre, *Scn2a*^fl/+^/*Vgat*-Cre and *Scn2a*^fl/+^ controls (*N* = 3, each genotype). Unpaired *t*-test, *Scn2a*^fl/+^ vs. *Scn2a*^fl/+^/*Emx1*-Cre, neocortex: *t*(4) = 5.91, **P = 0.0041; hippocampus: *t*(4) = 5.74, **P = 0.0046; *Scn2a*^fl/+^ vs. *Scn2a*^fl/+^/*Vgat*-Cre, neocortex: *t*(4) = 1.413, *P* = 0.2305; hippocampus: *t*(4) = 0.489, *P* = 0.6503. Nav1.2 protein was normalized by β-tubulin. Mean Nav1.2 expression levels are represented as percentages relative to the level of *Scn2a*^fl/+^ control littermates (100%). Data represent means ± SEM, **P < 0.01

**Fig. 7:**
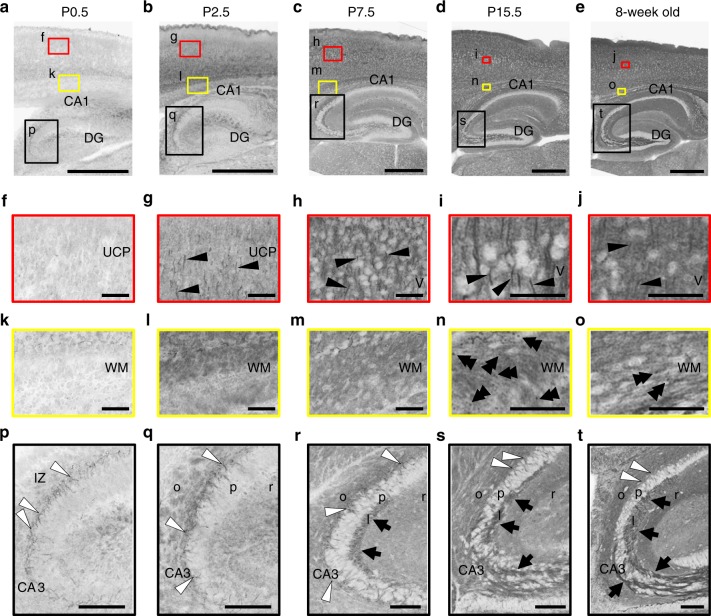
Developmental changes of Nav1.2 distribution in mouse brain. Brain sections of wild-type mice at P0.5 (**a**, **f**, **k**, **p**), P2.5 (**b**, **g**, **l**, **q**), P7.5 (**c**, **h**, **m**, **r**), P15.5 (**d**, **i**, **n**, **s**), and 8-week-old (**e**, **j**, **o**, **t**) were stained with anti-Nav1.2 (G-20, red). Higher-magnified images outlined in **a**–**e** are shown in **f**–**t**. Nav1.2 immunoreactivities were observed at AISs of neocortical neurons (single black arrowheads), nodes of Ranvier within white matter (double black arrowheads), AISs of hippocampal pyramidal neurons (white arrowheads), mossy fibers of dentate granule cells (black arrows), etc. Note that, while Nav1.2 at AISs and nodes of Ranvier peaked at P15.5 and became less at 8-weeks, diffused Nav1.2 signals in neocortex continued to become dense until 8-weeks-old. The brain slices were processed in parallel. Representative images of four or more slices per stage are shown. MZ marginal zone, UCP upper cortical plate, LCP lower cortical plate, DG dentate gyrus, WM white matter, IZ intermediate zone, o stratum oriens, p stratum pyramidale, l stratum lucidum, r stratum radiatum. Scale bars: **a**–**e** 500 µm; **f**–**o** 50 µm; **p**–**t** 100 µm

**Fig. 8:**
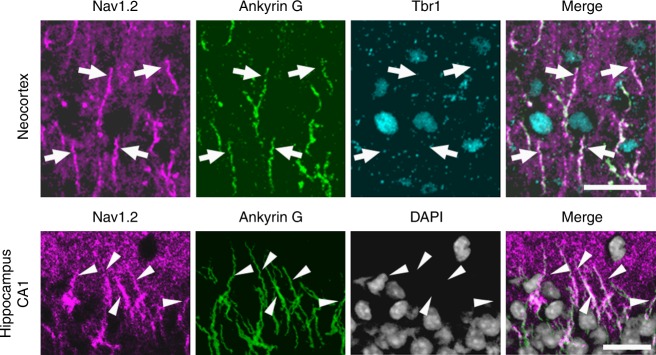
Nav1.2 expressions at AISs of excitatory neurons in neocortex and hippocampus. Immunofluorescence histochemistry of P15.5 wild-type neocortices and hippocampi stained with anti-Nav1.2 (G-20, magenta), anti-ankyrin G (green), and anti-Tbr1 (cyan) antibodies, and counterstained with 4′-6-diamidino-2-phenylindole (DAPI, gray) and their merged images. Arrows indicate Nav1.2 and ankyrin G-double immunoreactive AISs of Tbr1-expressing neocortical pyramidal cells. Arrowheads indicate Nav1.2 and ankyrin G-double immunoreactive AISs of hippocampal pyramidal cells. Representative images of four or more slices are shown. o stratum oriens, p stratum pyramidale. Scale bars: 20 µm

Western blot analyses showed that Nav1.2 expression was already detectable on embryonic day 14.5 and steeply up-regulated during postnatal development (Supplementary Fig. 4). At 6–8 weeks of age, Nav1.2 expression in 6-week-old neocortex and hippocampus showed ~50% reduction in *Scn2a*^fl/+^/*Emx1*-Cre and no significant alterations in *Scn2a*^fl/+^/*Vgat*-Cre, compared with *Scn2a*^fl/+^ mice (Fig. 6), indicating that most Nav1.2 is expressed in excitatory neurons in neocortex and hippocampus at adult stages. We further investigated the temporal and spatial expression pattern of Nav1.2 by immunohistochemistry. Although two commercial (ASC-002, G-20) and one original (EM-1) Nav1.2 antibodies displayed similar staining patterns (Supplementary Fig. 5) and their immunosignals disappeared in *Scn2a*^KO/KO^ when compared with *Scn2a*^+/+^ mice (Supplementary Fig. 6), the goat antibody G-20 gave the clear signal and allowed double staining with rabbit or mouse antibodies, and was selected for subsequent immunohistochemical analyses. At P0.5, immunosignals for ankyrinG were detected in both *Scn2a*^KO/KO^ and *Scn2a*^+/+^ mice, suggesting that AIS structures in *Scn2a*^KO/KO^ mice remain largely intact (Supplementary Fig. 7). Immunohistochemistry of Nav1.2 in wild-type mice detected immunosignals throughout the central nervous system with drastic changes in signal intensity and subcellular distribution from neonatal period to adulthood (Fig. 7 and Supplementary Figs. 4b and 8). During this period, the intensity of Nav1.2-immunosignals grew in unmyelinated fibers, including mossy fibers of hippocampal dentate granule cells (Fig. 7p–t) and cerebellar parallel fibers (Supplementary Fig. 8a-d). Nav1.2 signals also appeared and grew more intense in unmyelinated AIS segments of neocortical and hippocampal pyramidal cell axons (Fig. 7f–j, p–t) and nodes of Ranvier (Fig. 7k–o). At P15.5 in neocortex and hippocampus, Nav1.2 was strongly observed at the ankyrinG-positive AISs of excitatory neurons (Fig. 8). Consistent with previous studies^26,33^, the AISs of P15.5 neocortical pyramidal cells expressed Nav1.2-immunoreactivity in their proximal part and Nav1.6-immunoreactivity in their distal part (Supplementary Fig. 9). It is noteworthy that diffuse Nav1.2-immunoreactivity assumed to be distal axonal features throughout neocortex and hippocampus continued to become stronger until adulthood (Fig. 7), while such diffuse signals of Nav1.6 were not observed (Supplementary Fig. 10). These observations suggest that Nav1.2, rather than Nav1.6, is the major voltage-gated sodium channel at more distal axonal sites of excitatory neurons. To localize Nav1.2 in the cerebral cortex at adult stage, we employed pre-embedding silver-enhanced immunogold electron microscopy (Supplementary Fig. 11). Most metal particles binding Nav1.2 were detected on the cell membrane of thin processes, presumably distal unmyelinated portions of preterminal axon. By contrast, only a few particles were detected in axon terminals, and none at myelinated portions of axons.

### Reduced action potential amplitude in excitatory cells

Our previous study of voltage-clamp analysis of cultured *Scn2a*^KO/+^ hippocampal pyramidal neurons after 5–9 days in vitro showed a ~ 45% reduction in maximum sodium conductance density^38^. Here we further investigated the properties of action potentials of *Scn2a*^KO/+^ excitatory and inhibitory neurons in neocortical layer II/III and hippocampal CA1 region using current-clamp recordings of acute mouse brain slices. To discern between excitatory and inhibitory neurons, we crossed *Scn2a*^KO/+^ with a *Vgat*-Venus line that expresses green fluorescent proteins selectively in inhibitory neurons^44^.

**Fig. 9:**
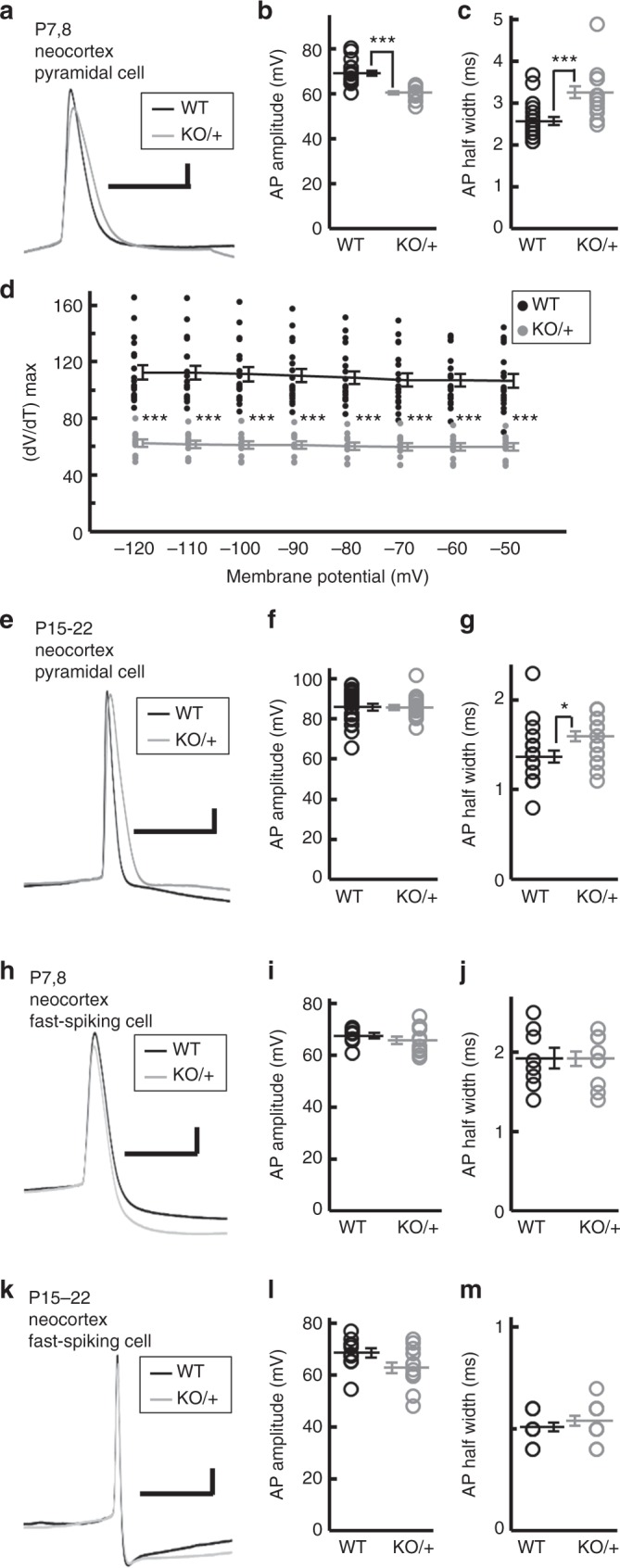
Altered action potentials of *Scn2a* KO excitatory neurons. Responses of neocortical pyramidal excitatory neurons and fast-spiking inhibitory neurons from *Vgat*-Venus/*Scn2a*^+/+^ (WT) mice (*n* = 19 pyramidal and 8 fast-spiking neurons, P7–8; *n* = 22 pyramidal and 11 fast-spiking neurons, P15–22) and *Vgat*-Venus/*Scn2a*^KO/+^ (KO/ + ) mice (*n* = 16 pyramidal and 11 fast-spiking neurons, P7–8; *n* = 22 pyramidal and 13 fast-spiking neurons, P15–22) to current injections. Representative action potential traces (**a**, **e**, **h**, **k**), peak amplitudes (**b**, **f**, **i**, **l**), half widths (**c**, **g**, **j**, **m**) and maximum rates of rise (**d**) were shown. **b**–**g** Peak amplitudes of P7–8 pyramidal cells are smaller in *Scn2a*^KO/+^ than in WT mice, while half widths of P7–8 and P15–22 pyramidal cells were broader in *Scn2a*^KO/+^ than in WT mice. Unpaired *t*-test, peak amplitudes, P7–8, *t*(33) = 6.0638, ***P = 0.0000008, P15–22, *t*(42) = 0.0791, *P* = 0.9373; half widths, P7–8, *t*(33) = −4.0342, ***P = 0.00031, P15–22, *t*(42) = −3.6442, *P = 0.0109. **d** Maximum rates of rise for action potential of P7–8 hippocampal pyramidal cells (*n* = 19 WT neurons, *n* = 11 KO/ + neurons) were lower in *Scn2a*^KO/+^ than in WT mice. Unpaired *t*-test, −120mV, *t*(28) = 7.3005, ***P = 0.00000006, −110mV, *t*(28) = 7.3572, ***P = 0.00000005, −100mV, *t*(28) = 7.3237, ***P = 0.00000006, −90mV, *t*(28) = 7.4814, ***P = 0.00000004, −80mV, *t*(28) = 7.6586, ***P = 0.00000002, −70mV, *t*(28) = 7.2738, ***P = 0.00000006, −60mV, *t*(28) = 7.8954, ***P = 0.00000001, −50mV, *t*(27) = 7.1199, ***P = 0.00000012. There were no significant differences between WT and *Scn2a*^KO/+^ fast-spiking neurons. Unpaired *t*-test, peak amplitudes, P7–8, *t*(17) = 0.8793, *P* = 0.3915, P15–22, *t*(22) = 2.0676, *P* = 0.0506; half widths, P7–8, *t*(17) = 0.0435, *P* = 0.9658, P15–22, *t*(22) = −0.8992, *P* = 0.3783. Details of the results and statistical tests are reported in Supplementary Tables 1 and 2. Black filled circles and gray filled circles represent WT and *Scn2a*^KO/+^ neurons, respectively. Data represent means ± SEM, *P < 0.01, ***P < 0.001. Scale bars: (**a**, **e**, **h**, **k**) vertical 10 mV; horizontal 10 milliseconds

In cortical pyramidal neurons at early postnatal ages (P7–8), peak amplitudes in single action potentials and spike trains were lower in *Scn2a*^KO/+^ than in *Scn2a*^+/+^ mice (Fig. 9a, b, Supplementary Fig. 12a, b and Supplementary Table 1). Half widths in single action potentials and spike trains were broader in *Scn2a*^KO/+^ than in *Scn2a*^+/+^ mice at P7–8 (Fig. 9a, c and Supplementary Table 1). Maximum rates of rise of action potential upstroke at threshold in response to different holding membrane potentials were lower in *Scn2a*^KO/+^ than in *Scn2a*^+/+^ mice at P7–8 (Fig. 9d), consistent with the lowered voltage-gated sodium channel current density in hippocampal cultured neurons from *Scn2a*^KO/+^ mice^38,45^. At P15–22, peak amplitudes in cortical pyramidal neurons were similar between the genotypes (Fig. 9e, f, Supplementary Fig. 12c, d and Supplementary Table 1), whereas half width was again broader in *Scn2a*^KO/+^ than in *Scn2a*^+/+^ (Fig. 9e, g and Supplementary Table 1). In hippocampal pyramidal cells, peak amplitudes were lower in *Scn2a*^KO/+^ than *Scn2a*^+/+^ mice at P7–8 but not at P15–20 (Supplementary Table 1). Half widths in single action potentials and spike trains were similar among the genotypes at both age groups (Supplementary Table 1). In contrast to these excitatory neurons, neocortical and hippocampal FS inhibitory neurons showed no significant differences in the electrophysiological properties between the genotypes at any age groups examined (Fig. 9h–m, Supplementary Fig. 13 and Supplementary Table 1). These results suggest that excitatory neural activity is primarily impaired whereas the FS inhibitory activity remains unchanged in *Scn2a*^KO/+^ mice.

## Discussion

Here we demonstrated ethosuximide-sensitive absence-like seizures with bilateral SWDs in mice heterozygous for a patient-derived *Scn2a*-RX nonsense knock-in mutation originally described in a patient with epileptic encephalopathy, intellectual disability, and ASD^4^. These same phenotypes were also observed in this study in a previously generated KO mutant mouse^38^. We further revealed that the RX mutation did not produce a truncated Nav1.2-RX peptide, but rather inactivated the mutated *Scn2a* allele, leading to Nav1.2 haplodeficiency in *Scn2a*^RX/+^ mice and presumably in the patient. Although we previously suggested a dominant-negative effect of Nav1.2-RX truncated protein^4^ and actually SWDs were rather prominent in *Scn2a*^RX/+^ mice compared to *Scn2a*^KO/+^ mice and itself may suggest a modifying effect of a minor amount of the Nav1.2 truncated protein which was not detectable in our western blot analysis, the incidence and duration of epileptiform discharges still did not significantly differ between *Scn2a*^RX/+^ and *Scn2a*^KO/+^ mice. This indicates that Nav1.2 haploinsufficiency is the major underlying basis for epileptic seizures. We further showed that a selective *Scn2a* deletion in dorsal-telencephalic excitatory neurons in mice (*Scn2a*^fl/+^/*Emx1*-Cre) reproduced the absence-like seizures, whereas mice with a global *Scn2a* deletion in inhibitory neurons (*Scn2a*^fl/+^/*Vgat*-Cre) showed no discernable epileptic abnormalities. Contrary to the suggestion that loss of functional Nav1.2 in inhibitory neurons may contribute to the pathogenesis of epileptic seizures in patients with *SCN2A* mutations^33^, our findings indicate that Nav1.2 haplodeficiency in excitatory neurons causes epilepsy.

*Scn2a*^fl/fl^/*Emx1*-Cre and *Scn2a*^fl/fl^/*Vgat*-Cre mice both died prematurely, suggesting that expression of Nav1.2 in either excitatory or inhibitory neurons is essential for postnatal viability. Nav1.2 expression in *Emx1*-Cre-positive excitatory neurons is robust in brain subregions, such as cerebral cortex, olfactory bulb and hippocampus^29^, and is estimated to explain ~30% of the total Nav1.2 amount in whole brain at P0.5. Similarly, the amount of Nav1.2 expressed in inhibitory neurons at P0.5 is estimated to be ~60% of the whole-brain Nav1.2 amount. We have shown that Nav1.2 is localized at AISs in vasoactive intestinal peptide-positive or reelin-positive/somatostatin-negative inhibitory neurons in neocortex and hippocampus, and distributed in unmyelinated axons of GABAergic medium spiny neurons in striatum^29,32^. Although the reason(s) for the premature death in these mice is unknown, given that perinatal death in *Scn2a*^KO/KO^ seems to be associated with severe hypoxia and massive neuronal apoptosis in brainstem^38^, Nav1.2 deficiency in cortico-brainstem projections or local inhibitory circuits in cerebral cortex and brainstem presumably contributes to premature death.

*SCN2A* mutations have been described in patients with a wide spectrum of epilepsies, intellectual disability and ASD. *SCN2A* mutations in patients with the severe end of epilepsies such as early-infantile epileptic encephalopathy, Ohtahara syndrome and West syndrome are almost exclusively missense, while nonsense, frameshift and splice site mutations are dominant in patients with ASD and intellectual disability associated with milder, later-onset epilepsy or without epilepsy (reviewed in Yamakawa^22^). Recent patch-clamp analyses confirmed that Nav1.2 channels with missense mutations found in patients with early-infantile severe epilepsies had gain-of-function effects, while mutations found in patients with ASDs or late-onset epilepsies had loss-of-function effects^35,36^. Although the epileptic phenotype of the patient with *SCN2A*-R102* mutation who showed intellectual disability and ASD was rather severe^4^, it was milder and later-onset compared to those of early-infantile epileptic encephalopathy. These observations suggest that *Scn2a*-deficient mice are models for ASD and intellectual disability with milder epilepsies rather than early-infantile epileptic encephalopathy, and that *Scn2a* knock-in mice with gain-of-function missense mutations are models for early-infantile epileptic encephalopathy. In fact, *Scn2a*^RX/+^ and *Scn2a*^KO/+^ mice did not show spontaneous convulsive seizures but only mild absence-like seizures with SWDs. Similarly, the patient carrying the *SCN2A*-R102* nonsense mutation showed absence and atonic seizures^4^. The patient with ASD and intellectual disability harboring the splice site mutation, putatively *SCN2A*-K90Vfs*9, was also reported to have behavioral episodes characterized by a stone faced expression and limp posture, suggestive of absence seizures^11^. Absence or absence-like epilepsies in patients with ASD and intellectual disability is consistent with absence-like seizures in mice with *Scn2a*-haploinsufficiencies.

The *Scn2a*^Q54^ mouse, long considered a model of diseases with *SCN2A* gain-of-function mutations, showed partial seizures originating from hippocampus^37^. In contrast, our ECoG recordings of *Scn2a*^RX/+^, *Scn2a*^KO/+^, and *Scn2a*^fl/+^/*Emx1*-Cre mice all showed absence-like seizures with SWDs. It is plausible that loss-of-function and gain-of-function *Scn2a* mutations culminate in different seizure phenotypes with specific patterns of epileptiform discharges. However, Nav1.2 expression in the *Scn2a*^Q54^ mouse was driven by the neuron-specific enolase promoter and it may lead to ectopic expression and other epistatic effects. Direct comparisons with knock-in mice bearing *Scn2a* missense mutations found in patients with early-infantile epileptic encephalopathies under the control of intrinsic *Scn2a* promoters may provide a more accurate model.

Febrile seizures are highly unusual for patients with *SCN2A* mutations^2–5^, despite the proposed role of Nav1.2 in febrile seizure generation^46,47^. We showed that *Scn2a*^KO/+^ mice exhibit normal susceptibility to hyperthermia-induced seizures that seems reminiscent of the temperature-independent nature of the epilepsies in a major proportion of the patient population. This contrasts with the temperature-sensitivity of epilepsies in patients with *SCN1A* mutations^48,49^ and *Scn1a*-deficient mice^50,51^^,^ and rats^52^. Such disparate temperature-sensitivities of seizures in patients and animal models harboring *SCN1A* and *SCN2A* mutations may be accounted for by the distinct cellular and regional distributions for Nav1.1 and Nav1.2 rather than by distinct kinetics of the proteins^22,29^.

Our immunohistochemistry data showed that at unmyelinated fibers such as hippocampal mossy fibers, axons of striatal medium spiny neurons and parallel fibers of cerebellar granule cells, Nav1.2 is expressed and incremented through development. In contrast, Nav1.2 at AISs and nodes of Ranvier in myelinated fibers in neocortex and hippocampus became intense at ~P15 where Nav1.2 and Nav1.6 were co-expressed at proximal and distal AISs respectively. Nav1.2 density in these areas gradually decreased in later stages and was replaced with Nav1.6, consistent with previous studies^26–28,30–32,53^. However, we found that diffuse Nav1.2 signals in neocortex and hippocampus (presumably in synaptic terminals) continued to increase through development. Of note, Nav1.2 was suggested to be abundantly expressed in synaptic terminals of cerebellar parallel fibers^54^. Contrary to the increase of the diffuse Nav1.2 signals, Nav1.6 did not show such increase in the corresponding brain regions, suggesting that Nav1.2 is a major voltage-gated sodium channel in axon terminals responsible for synaptic transmission at adult stages. Because of rather late onset of epilepsies in patients with *SCN2A* loss-of-function mutations and the reproduction of absence seizures in mice with dorsal-telencephalic excitatory neuron-specific *Scn2a* deletion, it would be now of interest whether the seizures are caused by Nav1.2 deficiency at the distal axons of neocortical or other dorsal-telencephalic excitatory neurons.

Our whole-cell current-clamp recordings showed that, at early postnatal stage (P7–8), neocortical and hippocampal *Scn2a*^KO/+^ excitatory pyramidal neurons displayed decreased action potential peak amplitudes by ~10–15%. Nevertheless, our previous study using voltage-clamp recordings showed a ~45% reduction in maximum sodium conductance density in dissociated hippocampal neurons from *Scn2a*^KO/+^ newborn mice after 5–9 days culture in vitro^38^. Similarly, acutely dissociated guinea-pig hippocampal neurons treated with a moderate dose of tetrodotoxin, a sodium channel blocker, reduced sodium current to less than half, whereas action potential amplitude was only slightly affected, suggesting a surplus of sodium channels in neurons for action potential firing^55^. However, the study showed that such surplus was required for repetitive action potential firings, and this could also be the case in *Scn2a*^KO/+^ mice. The present current-clamp recordings further showed that action potential peak amplitude recorded from neocortical and hippocampal *Scn2a*^KO/+^ excitatory neurons reached normal levels at P15–22, raising a possibility that Nav1.2 may not be needed to generate action potentials at later postnatal stage, consistent with the developmental changes in subcellular localization and distribution of Nav1.2. Alternatively, Nav1.2 haploinsufficiency may be compensated by other subtypes of voltage-gated sodium channel. No significant changes in mRNA expression levels of the other sodium channel subunit genes were observed in *Scn2a*^KO/+^ brains, excluding dosage compensation of loss of Nav1.2. Instead, Nav1.6 co-expression with Nav1.2 in the AISs of pyramidal neurons may compensate for loss of Nav1.2. Steep up-regulation of Nav1.6 expression in the AISs of pyramidal neuron between P7.5 and P15.5 could account for the significant decrement of action potential peak amplitudes in *Scn2a*^KO/+^ pyramidal neurons at P7–8, but not at P15–22. Despite the possible functional compensation for loss of Nav1.2 at AISs and nodes of Ranvier, Nav1.6 may not have sufficient compensatory effects on absence-like seizures at adult stages, presumably due to the limited expression of Nav1.6 at synaptic terminals. It has been reported that impairing voltage-gated sodium channels function in FS parvalbumin-positive inhibitory neurons in mice led to epileptic seizures^25^. However, no differences were detected in the responses of neocortical FS interneurons to current injections between *Scn2a*^KO/+^ and wild-type mice, consistent with our previous observation that Nav1.2 is not expressed in parvalbumin-positive interneurons^29^.

How does the Nav1.2 haploinsufficiency in dorsal-telencephalic excitatory neurons cause epileptic seizures? One possible mechanism is abnormal activity in thalamocortical circuits, which has long been proposed as a basis for absence epilepsy^56,57^. Impaired firing properties in cortical excitatory neurons may impact their downstream input onto inhibitory neurons in the thalamic reticular nucleus, which in turn fails to suppress excitatory thalamocortical relay neurons. The excited thalamocortical neurons may then provide feedback inhibition through the thalamic reticular nucleus inhibitory neurons and generate thalamocortical hyper-synchronous oscillations that result in absence seizures^57^. P/Q-type calcium channel gene *CACNA1A* mutations have been described in patients with absence epilepsy^58^, and *Cacna1a* KO mice have absence epilepsies^41^. Recently, we reported that a selective *Cacna1a* gene deletion in cortical layer VI pyramidal cells, which innervate thalamic relay neurons and reticular thalamic neurons, caused upregulation of T-type calcium current in thalamic relay neurons and resulted in absence epilepsy in mice^59^, suggesting that impaired cortical excitatory input to thalamic regions may cause rebound burst of thalamocortical relay neurons and leads to thalamocortical hyper-synchronous oscillations as may be the case for ethosuximide-sensitive absence-like seizures in *Scn2a*-deficient mice. A second plausible mechanism considers a rebound hyper-excitability within neocortex. In neocortex, excitatory, and inhibitory neurons form reciprocal and highly complex networks. Impaired excitatory inputs into inhibitory neurons may even disinhibit the downstream excitatory neurons and the consequent epileptic discharges widely spread to the cortico-thalamic circuit. Meeren and colleagues^60^ actually reported that somatosensory cortex is the initiation site of epileptic activity in a rat model of absence epilepsy. Thirdly, the predominant appearance of epileptiform discharges in CPu of *Scn2a*^KO/+^ mice and *Scn2a*^fl/+^/*Emx1*-Cre mice may implicate CPu in the pathology of absence-like seizures. CPu is the largest compartment of basal ganglia that receive excitatory inputs from cortex and thalamus and send output back to the thalamus, and cortex via thalamus. Basal ganglia were suggested to modulate the occurrence of SWDs generated in the thalamocortical circuits^61^. The present ECoG recordings also revealed an increased incidence of SWDs in *Scn2a*^fl/+^/*Emx1*-Cre mice, compared to *Scn2a*-haploinsufficient mice. We surmise that the remaining Nav1.2 expression in non-dorsal telencephalic regions such as CPu^32^ of *Scn2a*^fl/+^/*Emx1*-Cre mice may have aggravating effects on absence-like seizures. Alternatively, given Nav1.2 expression in caudal ganglionic eminence-derived vasoactive intestinal peptide- or reelin-positive inhibitory neurons of neocortex and hippocampus^29^, selective Nav1.2 elimination in excitatory neurons may shift an excitation/inhibition balance toward inhibition, which enhances hyperpolarization of neurons and low threshold rebound burst-firing via de-inactivation of T-type calcium currents, increasing the risk of SWD generation. Furthermore, it is possible that the decreased neuronal activity caused by the Nav1.2 deficit affects network formation during development. Congenital *Scn2a* mutations may impair or affect maturation, migration, or innervations of inhibitory or excitatory neurons and alter global brain network excitability. Thus, the circuit basis for the development of absence-like seizures in mice with *Scn2a*-deficiency requires further study.

In summary, we showed that *Scn2a*-haploinsufficiency in mice gives rise to absence-like seizures in a dorsal telencephalic excitatory neuron-dependent manner. Our findings should contribute to understanding of the pathomechanisms of epilepsies in patients with *SCN2A* mutations and absence epilepsy itself, the mechanism of which is still not fully elucidated.

## Methods

### Animals

All animal experimental protocols were approved by the Animal Experiment Committees of RIKEN Institute and Shizuoka Institute of Epilepsy and Neurological Disorders.

### Generation of knock-in *Scn2a* mice with the R102* mutation

We isolated the PAC clones 348A2 and 386F4 by screening a pooled mouse genomic PAC library (BACPAC Resource Center, Oakland, CA, USA) with dot blot hybridization using [α-32P] dCTP-labeled DNA corresponding to the genomic fragment containing exon 2 of the mouse *Scn2a* gene as the probe. A 400 bp *Pma*CI fragment of a PAC clone was inserted into the blunt-end filled *Bgl*II site of pEGFP-C2 (TaKaRa Bio, Shiga, Japan) to obtain pL1. Next, a 5 kb *Eco*105I-*Afl*III fragment of a PAC clone was inserted into the *Afl*III and blunt-end filled *Sac*I sites of pL1 to generate pL2. Then, a *Sal*I fragment of pMCDTApA (a generous gift from Dr. Yagi, Osaka University) was inserted into the *Xho*I site of pL2 to generate pL3. In order to obtain pR1, a 3.2 kb *Pma*CI-*Eco*RI fragment of a PAC clone was inserted into the *Eco*RI and blunt-end filled *Hind*III sites of pEGFP-C2. Using the QuikChange Site-Directed Mutagenesis kit (Agilent Technologies, Santa Clara, CA, USA), the nucleotide substitution (CGG to TGA) leading to the R102* (RX) mutation was introduced into pR1. Following that, a 2.0 kb *Eco*RI-*Eco*RV fragment of a PAC clone was inserted into the *Eco*RI and blunt-end filled *Sal*I sites of pR1mutated to obtain pR2. A 1.2 kb *Eco*RI fragment of a PAC clone was subsequently inserted into the *Eco*RI site of pR2 to generate pR3. In order to inactivate the *Sac*II site in pR3, pR3 was digested with *Sac*II, filled with T4 DNA polymerase and self-ligated. Then, an *Xho*I-*Apa*I fragment of the resulted plasmid vector was inserted into the *Xho*I and *Apa*I sites of ploxPfrtPGKneofrt to generate pR4. Finally, pR4 was digested with *Sac*II and *Not*I and was inserted with a *Sac*II-*Eco*52I fragment of pL3 to create the targeting vector. All constructs were verified by sequencing.

The targeting vector was digested with *Sac*II for linearization and transfected into E14 ES cells with a Gene-Pulser (Bio-Rad, Hercules, CA, USA) at 3 µF and 800 V. Transfected ES cells were placed on neomycin-resistant, mitomycin C-treated mouse embryonic feeder cells, and neomycin-resistant ES colons were selected in the presence of 150 µg mL^−1^ of Geneticin (G418, Thermo Fisher Scientific, Waltham, MA, USA). *Eco*RI-digested genomic DNA was isolated from individual clones and analyzed by Southern blotting using the 5′ and 3′ probes that corresponded to the genomic sequence upstream and downstream of the targeting vector. PCR analysis was performed for verifying the presence of the RX mutation. The ES cells from two correctly targeted clones (7A1 and 9H1) were injected into C57BL/6J blastocysts to produce male chimeras with greater than 50% agouti coat color, which were then bred to C57BL/6J females to obtain F1 mice heterozygous for the RX mutation. F1 heterozygotes were subsequently crossed with C57BL6 mice to generate F2 *Scn2a*^RX+neo/+^ mice that were crossed with *CAG*-Flpe transgenic mice in a C57BL/6J background^62^ to remove the neo cassette. The absence of the neo cassette in F3 *Scn2a*^RX/+^/*CAG*-Flpe mice was verified by PCR. F3 *Scn2a*^RX/+^/*CAG*-Flpe mice were then crossed with C57BL/6J mice to obtain Flpe-lacking F4 *Scn2a*^RX/+^ mice. Homozygous mice were obtained by interbreeding F4 *Scn2a*^RX/+^ mice. No phenotypic differences were observed among the mice derived from the two ES cell clones. *Scn2a*^fl/+^ mice were thereafter maintained by crossing with C57BL/6J mice. *Scn2a*^RX/+^ mice on a congenic C57BL/6J background for more than 10 backcross generations were subjected to ECoG-EMG recordings. Genotyping of *Scn2a* knock-in mice was performed by PCR with the specific primers (forward: 5′- TGT CTC AGA TCC CCT ATT GCT -3′, reverse: 5′- CTT GGT AAC TTT GCC GAG TC -3′) that detect the wild-type allele (269 bp) and the targeted knock-in allele (484 bp) (Supplementary Fig. 1c). Unprocessed original scans of blots/gels are shown in Supplementary Fig. 14.

### *Scn2a* knockout mice

*Scn2a* KO mouse line has been generated by an insertion of neo cassette into exon 1 of the *Scn2a* gene^38^. *Scn2a*^KO/+^ mice were maintained on a congenic C57BL/6J background for more than 10 backcross generations.

### Generation of *Scn2a* conditional knockout mice

The targeting vector harboring the floxed allele was generated from the plasmid carrying the RX allele by correcting the gene mutation in coding exon 2 and inserting two loxP cassettes on either side of exon 2 with the Red/ET recombination system (Gene Bridges, Dresden, Germany). Briefly, a genomic fragment containing wild-type exon 2 was amplified by PCR with primers having *Hin*dIII sites at their 5′ ends, whose nucleotide sequences were as follows: 5′- CCC AAG CTT CGT GTA AGG GGA AAA AGT TCT A -3′ and, 5′- CCC AAG CTT AAG TGT TGA AGG GAG TGA GTG A -3′. The resultant amplicons were digested with *Hin*dIII and inserted into the *Hin*dIII site of pfrt-PGK/gb2-neo/kan-frtloxP to generate pfrt-PGK/gb2-neo/kan-frtloxP+exon2. Next, a genomic fragment containing downstream of exon 2 was amplified by PCR with primers having *Xho*I sites at their 5′ ends, whose nucleotide sequences were as follows: 5′- TTT TCT CGA GCA TTC ACT TTA GTG AGA TGG C -3′ and, 5′- TTT TCT CGA GAA AGT CCA GTG CAT GTA TG -3′. The resultant PCR products were digested with *Xho*I and inserted into the *Xho*I site of pfrt-PGK/gb2-neo/kan-frtloxP+exon2. The resulting plasmid DNA was then amplified by PCR with primers: 5′- CAT TCT GCA CGC TTC AAA G -3′ and, 5′- CAC CAT AAA GCT CAA AGG CA -3′. The PCR amplicons were used for transformation of an *Escherichia coli* strain, containing the targeting vector carrying the R102* allele and pSC101-BAD-gbaA (Gene Bridges). The resulting targeting vector harboring the floxed allele was verified by restriction enzyme digestion and DNA sequencing.

The targeting vector harboring the floxed allele was digested with *Sac*II for linearization and transfected into E14 ES cells with a Gene-Pulser (Bio-Rad). The ES cells from two correctly targeted clones (2B2 and 3H12) were injected into C57BL/6J blastocysts to obtain male chimeras that were subsequently bred to C57BL/6J females to generate F1 mice heterozygous for the targeted allele. F1 heterozygotes were then crossed with *CAG*-Flpe transgenic mice on a C57BL/6J background^62^ to generate N2 mice heterozygous (*Scn2a*^fl/+^/*CAG*-Flpe) for the floxed allele and lacking the neomycin cassette. Absence of the neo-cassette was verified by PCR analysis. *Scn2a*^fl/+^/*CAG*-Flpe mice were subsequently crossed with C57BL/6J mice to obtain N3 *Scn2a*^fl/+^ mice without the *CAG*-Flpe transgene. Absence of the *CAG*-Flpe transgene was verified by PCR analysis. *Scn2a*^fl/+^ mice were thereafter maintained by crossing with C57BL/6J mice. Homozygous (*Scn2a*^fl/fl^) mice were obtained by interbreeding *Scn2a*^fl/+^mice. No phenotypic differences were observed among the mice derived from the two ES cell clones. Genotyping of *Scn2a* conditional KO mice was performed by PCR with the specific primers (forward: 5′- TGT CTC AGA TCC CCT ATT GCT -3′, reverse: 5′- CCA GTA GAA CAC CAT AAA GCT CA -3′) that detect the wild-type allele (925 bp), the targeted floxed allele (1,162 bp) and the deleted allele (284 bp) (Supplementary Figs. 2c and 3a). Unprocessed original scans of blots/gels are shown in Supplementary Fig. 14.

### EIIa-Cre, *Emx1*-Cre, *Vgat*-Cre, and *Vgat*-Venus mouse lines

The EIIa-Cre transgenic line, in which the Cre-loxP recombination occurs in germline cells, was previously generated by injection of the Cre cassette under the control of the adenovirus EIIa promoter into mouse zygotes^42^, and was maintained on a C57BL/6J background. EIIa-Cre mice were cross-mated with *Scn2a*^fl/fl^ mice, and heterozygous (*Scn2a*^fl/+^/EIIa-Cre) offspring were subsequently backcrossed with C57BL/6J mice to obtain *Scn2a*^del/+^ mice lacking the EIIa-Cre transgene. Absence of the EIIa-Cre transgene was verified by PCR analysis. Homozygous (*Scn2a*^del/del^) mice were obtained by interbreeding *Scn2a*^del/+^ mice.

*Empty spiracles homolog 1* (*Emx1*)-Cre knock-in line without a neomycin cassette was previously generated by targeted insertion of the Cre cassette into the *Emx1* gene^43^, and was maintained on a C57BL/6J background. Heterozygous (*Scn2a*^fl/+^/*Emx1*-Cre) mice were obtained by cross-mating *Scn2a*^fl/fl^ mice and *Emx1*-Cre mice, and subsequently backcrossed with *Scn2a*^fl/fl^ mice to obtain homozygous (*Scn2a*^fl/fl^/*Emx1*-Cre) mice.

*Vesicular GABA transporter* (*Vgat*, also known as vesicular inhibitory amino acid transporter, *Viaat*)-Cre BAC transgenic line was previously generated by pronuclear injection of the Cre cassette under the control of the mouse *Vgat* promoter^25^, and maintained on a C57BL/6J background. Heterozygous (*Scn2a*^fl/+^/*Vgat*-Cre) mice were obtained by cross-mating *Scn2a*^fl/fl^ mice and *Vgat*-Cre mice. Because the *Scn2a* and *Vgat*-Cre transgene alleles were mapped close together on the same chromosome^25^, the floxed *Scn2a* and *Vgat*-Cre alleles in *Scn2a*^fl/+^/*Vgat*-Cre obtained by cross-mating *Scn2a*^fl/fl^ mice and *Vgat*-Cre mice were presumed to be arranged in trans-configuration. *Scn2a*^fl/+^/*Vgat*-Cre mice were then bred with C57BL/6J mice to obtain *Scn2a*^fl/+^/*Vgat*-Cre mice, in which the floxed *Scn2a* and *Vgat*-Cre transgene alleles were located in cis-configuration as a result of germ-line recombination between the alleles in *Scn2a*^fl/+^/*Vgat*-Cre parents. The resultant *Scn2a*^fl/+^/*Vgat*-Cre offspring were subsequently crossed with *Scn2a*^fl/fl^ mice to obtain homozygous (*Scn2a*^fl/fl^/*Vgat*-Cre) mice, whereas *Scn2a*^fl/+^ offspring were crossed with *Scn2a*^fl/fl^ mice to obtain *Scn2a*^fl/fl^ controls

Genotyping of EIIa-Cre, *Emx1*-Cre and *Vgat*-Cre mice was performed by PCR with the Cre-specific primers (forward: 5′- AGG TTC GTT CAC TCA TGG A- 3′, reverse: 5′- TCG ACC AGT TTA GTT ACC C -3′) that yield a 235-bp amplicon.

*Vgat*-Venus transgenic mouse line expressing a fluorescent protein specifically in inhibitory cells has been generated by pronuclear injection of the Venus cassette under the control of the mouse *Vgat* promoter^44^.

### Antibody generation

Rabbit polyclonal anti-N-terminus-Nav1.2 antibody (EM-1) was raised against oligopeptides corresponding to the amino acids MAQSVLVPPGPDSFRFF of mouse Nav1.2 plus C at its C-terminus for coupling. Antibody was then affinity-purified using the SulfoLink kit (Thermo Fisher Scientific). Nav1.2-immunoreactivity was abolished when the antibody was preabsorbed with the immunogenic oligopeptides.

### Western blot analyses

Brains were obtained from mice (P0.5 *Scn2a*^RX/RX^, *Scn2a*^RX/+^ and *Scn2a*^+/+^ littermate mice: 6-week-old *Scn2a*^fl/+^/*Emx1*-Cre, *Scn2a*^fl/+^/*Vgat*-Cre and *Scn2a*^fl/+^ littermate mice: P0.5 *Scn2a*^fl/fl^/*Emx1*-Cre, *Scn2a*^fl/fl^/*Vgat*-Cre and *Scn2a*^fl/fl^ control mice: both sex, C57BL/6J congenic background, P21.5 *Scn2a*^fl/fl^ and *Scn2a*^+/+^ littermate mice: P0.5 *Scn2a*^del/del^, *Scn2a*^del/+^, and *Scn2a*^+/+^ littermate mice: both sex, C57BL/6J and 129 mixed background), and homogenized in homogenization buffer (0.32 M sucrose, 10 mM HEPES, 2 mM EDTA and 1X complete protease inhibitor cocktail (Roche Diagnostics, Indianapolis, IN, USA), pH 7.4, and centrifuged for 15 min at 1000 × *g*. The supernatants were next centrifuged for 30 min at 30,000 × *g*. The resulting supernatants were designated as the total brain cytosol fraction. The pellets were subsequently resuspended in lysis buffer (50 mM HEPES and 2 mM EDTA, pH 7.4) and centrifuged for 30 min at 30,000 × *g*. The resulting pellets, designated as the total brain membrane fraction, were dissolved in 2 M Urea, 1X NuPAGE reducing agent (Thermo Fisher Scientific) and 1X NuPAGE LDS sample buffer (Thermo Fisher Scientific). Total brain cytosol and membrane fractions were separated on the NuPAGE Novex Tris-acetate 3–8% gel (Thermo Fisher Scientific) or the PAG mini SuperSep Ace Tris-glycine 5–20% gel (Wako, Tokyo, Japan), transferred to a nitrocellulose membrane (Bio-Rad) and immunoblotted. Blots were probed with the rabbit anti-N-terminus Nav1.2 (~ 200 ng mL^−1^; EM-1), rabbit anti-internal-region Nav1.2 (1:200; ASC-002, Alomone, Jerusalem, Israel), goat anti-internal-region Nav1.2 (1:200; SC-31371, G-20, Santa Cruz Biotechnology, Santa Cruz, CA, USA), rabbit anti-internal-region Pan Nav1 (1:1,000; SP19)^23^, and mouse anti-β tubulin (1:10,000; T0198, Sigma-Aldrich, St Louis, MO, USA) antibodies. The blot was then incubated with horseradish peroxidase-conjugated goat anti-mouse IgG (1:5000; W4011, Promega, Madison, WI, USA), rabbit anti-goat IgG, Fc fragment specific (1:10,000; 305-035-046, Jackson ImmunoResearch, West Grove, PA, USA) or goat anti-rabbit IgG (1:2,000; SC-2004, Santa Cruz Biotechnology,) antibody and bound antibodies were detected using the enhanced chemiluminescence reagent (PerkinElmer, Boston, MA, USA). Semi-quantitation of proteins was performed using the NIH ImageJ software (National Institutes of Health, Bethesda, MD, USA). Means signal intensities were quantified in each genotype and represented as percentages relative to wild-type littermates. Mean expression levels were estimated by comparison with serial dilutions of homogenates from age-matched control mice and represented as percentages relative to control mice. Unprocessed original scans of blot films are shown in Supplementary Figs. 15–18.

### Electrocorticography-electromyography recordings

Adult mice (6–11-week-old *Scn2a*^RX/+^ and *Scn2a*^+/+^ littermate mice: 10–27-week-olds *Scn2a*^KO/+^ and *Scn2a*^+/+^ littermate mice: 6–8-week-old *Scn2a*^fl/+^/*Emx1*-Cre, *Scn2a*^fl+^/*Vgat*-Cre and control littermates: both sex, C57BL/6J congenic background) were used in this study. The ECoG electrodes were implanted using 1.5% halothane anesthesia with N_2_O:O_2_ (1:1) ventilation. Stainless-steel screws (1.1 mm diameter), and in some experiments, Teflon-insulated silver wires served as ECoG electrodes and were secured to the skull and dura over the somatosensory cortex (1.5 mm lateral to midline, 1.0 mm posterior to bregma) and the cerebellum (at midline, 2.0 mm posterior to lambda) as a reference electrode. The EMG electrodes were placed 2–3 mm apart from the cervical region of the trapezius muscle. Recordings were obtained at least 1 week after the electrodes were implanted, to allow for full recovery. Video monitoring was simultaneously performed to detect behavioral seizures. Recordings were performed for a week, sampled at 500 Hz and then analyzed off-line using a software, SleepSign (Kissei Comtec, Nagano, Japan), blind to the genotype.

Ethosuximide (33.3 mg mL^−1^ in saline, 200 mg per kg body weight, Toronto Research Chemicals), or vehicle (the same volume of saline) was intraperitoneally injected.

### Local field potential recordings from behaving mice

Adult mice (6–8-week-old *Scn2a*^KO/+^ and control littermates: 3–6-month-old *Scn2a*^fl/+^/*Emx1*-Cre and control littermates: both sex, C57BL/6J congenic background) were used. A stainless steel screw (1.1 mm diameter) served as ECoG electrode was placed over the somatosensory cortex (1.0 mm posterior to bregma, 1.5 mm right to the midline) under Nembutal (50 mg per kg) and 1.5% halothane anesthesia with N_2_O:O_2_ (3:2) ventilation. A stainless screw as a reference and ground electrode was placed on the cerebellum. To record LFPs of brain regions, linear insulated stainless wires (200 μm diameter) with beveled tip were implanted ipsilaterally according to the following coordinates (from bregma, from midline, depth from the cortical surface): prefrontal cortex (1.9, 0.3, 1.4), caudate-putamen (0.0, 2.4, 2.5), basolateral amygdala (−1.4, 2.9, 3.7), ventroposterior thalamus (−1.8, 1.5, 3.2), hippocampus CA1 (−2.5, 2.2, 1.1), visual cortex binocular zone (−3.4, 3.0, 0.4). Contacts between the electrodes and brain tissue was covered with a small amount of Vaseline and secured with dental acrylic. An antibiotic (ampicillin) was used in surgery. LFPs (filtered 0.7–70 Hz, 1 kHz sampling) were recorded using the MAP data acquisition system (Plexon, Dallas, TX, USA) and analyzed off-line using a software, NeuroExplorer 4 (Nex Technologies, Madison, AL, USA)^63^.

ECoG and LFP signals with 3 or more high-amplitude-spikes that were over twice more than that of the background signal in a 1-s window, were considered to be epileptiform discharges and included in the analysis.

### Measurement of pentylenetetrazol-induced seizures

Adult mice (10-week-old *Scn2a*^RX/+^, *Scn2a*^KO/+^ and *Scn2a*^+/+^ littermates, both sex, C57BL/6J congenic background) were used. Experiments of PTZ injection were performed between 9:00 a.m. and 6:00 p.m. A freshly prepared PTZ (Sigma-Aldrich) solution was administered intraperitoneally at a dosage of 25 or 50 mg per kg body weight in a total volume of 100–250 μL. After PTZ injection, the mice were placed in a clear plastic cage and observed for 30 min or earlier if the animal died before 30 min. Video monitoring system was used to determine the latency to the first behavioral seizures from the time of PTZ injection. The behavioral seizure events were determined based on the behavioral and motor activities. We classified the seizures as absence seizure-like immediate behavioral arrest, myoclonic, clonic, and generalized tonic-clonic seizures. Absence-like seizures consisted of > 2 sec behavioral arrest with or without vibrissal and facial myoclonus. Myoclonic seizures consisted of body twitching. Clonic seizures consisted of a whole body jerk with or without irregular, bilateral forelimb movements. Generalized tonic-clonic seizures consisted of fully generalized clonic seizures sometimes followed by a tonic phase involving entire body and result in death. In calculations, 1800 s were assigned for mice without seizures 30 min after PTZ administration. Latency of behavioral seizures by PTZ injection was determined in a genotype-blinded manner.

### Measurement of hyperthermia-induced seizures

Mice (4-week-old *Scn2a*^KO/+^ and *Scn2a*^+/+^ littermates, male, C57BL/6J congenic background) were put on the perforated horizontal partition with 9 × 9 holes with diameter of 2.5 mm in a hermetically-enclosed plexiglass box (30 × 30 × 30 cm) and heated by blowing hot air from below using Air-Therm (World Precision Instruments, Sarasota, FL, USA). Rectal temperature was continually monitored at baseline and at seizure onset by temperature probe (IT-18; Physitemp Instruments, Clifton, NJ, USA). Before increment of air temperature, mouse was kept at least 3 min at 37 °C. We gradually elevated the body temperature by 0.5 °C per 2 min. When a seizure appeared, the mouse was promptly rescued by cooling with ice cubes. Measurement of the body temperature threshold in which seizure was induced was made.

### Quantitative RT-PCR

Brains were obtained from mice (P14.5 *Scn2a*^KO/+^ mice and *Scn2a*^+/+^ littermates, both sex), and total RNA was isolated and purified from brain using the RNeasy Midi Kit (Qiagen, Valencia, CA, USA), and reverse transcribed using the Super Script III First Strand Synthesis System (Thermo Fisher Scientific). The resultant cDNA was then amplified using the Applied Biosystems 7500 Real-Time PCR System (Thermo Fisher Scientific) with pre-designed TaqMan assays for mouse *Scn1a*, *2a*, *3a*, *5a*, *8a*, and *1b*-*4b* as well as the internal standard *18* *S rRNA* or *GAPD* (Thermo Fisher Scientific). Relative gene expression was determined using the 2^−ΔΔCt^ method^64^. Briefly, the cycle threshold value (Ct) on each PCR amplicon curve was determined, and the ΔCt was calculated by subtracting the Ct value of the internal standard from the Ct value of sodium channel mRNA. The ΔΔCt was then calculated by subtracting the ΔCt value for each individual from the mean ΔCt value of controls. Relative gene expression was calculated as 2^−ΔΔCt^ and represented as percentages, relative to mean expression level of *Scn2a*^+/+^ littermate controls (100%).

### In vitro electrophysiological recording

Current-clamp recordings were made from pyramidal or FS cells in visual cortex (P7–23) or hippocampus (P7–22) of both *Vgat*-Venus/*Scn2a*^+/+^ and *Vgat*-Venus/*Scn2a*^KO/+^ mice (both sex, C57BL/6J congenic background). Mice were deeply anesthetized with isoflurane. After decapitation, coronal slices (350 μm) from mice were cut in ice-cold dissection buffer containing (in mM): 225 sucrose, 2.5 KCl, 1.25 NaH2PO4, 10 MgSO4, 0.5 CaCl2, 26 NaHCO3, 10 glucose, bubbled with 95% O2, 5% CO2 (pH 7.4). Slices were incubated in normal artificial cerebrospinal fluid (ACSF)(in mM): 125 NaCl, 2.5 KCl, 1.25 NaH2PO4, 1 MgSO4, 2 CaCl2, 26 NaHCO3, 10 glucose, 3 sodium pyruvate and 1 ascorbate, at least for 1 h at 35 °C before recording. Recordings were made in a chamber superfused at 3 ml/min with the same ACSF maintained at 30–31 °C. Whole-cell patch-clamp recordings were obtained from layer II/III pyramidal cells or FS interneurons and using electrodes (4–5 MΩ) filled with an internal solution containing (in mM): 126 K-gluconate, 8 KCl, 2 NaCl, 0.2 EGTA, 20 HEPES, 4 MgATP, 0.3 Na3GTP, 14 phosphocreatine, and 0.1 Alexa Fluor 594 hydrazide. To visualize interneurons, we crossed *Scn2a* mice with *Vgat*-Venus transgenic mice and selected FS-interneurons from among all types based on their high frequency firing rate. Data were filtered (2 kHz), digitized (10 kHz), stored, and analyzed using pCLAMP 10 and Origin 8.5J software. To assess membrane properties at resting potentials, pyramidal cells or FS-interneurons were injected with negative square-wave current pulses (500 msec) repeated five times at 10 s intervals. To assess the properties of single action potential, cells were hyperpolarized to −120 mV from resting potential for 500 msec to induce maximal inactivation state for sodium channels, and then stepped square pulses every 4 pA were injected every 15 sec for triggering a single action potential. Firing frequency and adaptation were evaluated as multiples of threshold intensity for triggering a single action potential. To calculate dV/dt-max of each membrane potential from −120 to −60 mV, cells were first held at −50 mV, and then hyperpolarized at each potential for 1 s. Immediately after the end of hyperpolarization, cells were depolarized to generate action potentials by applying the current injection.

### Immunohistochemistry and immunofluorescence histochemistry

Immunohistochemistry was carried out as previously described^29^. Briefly, mice (P0.5, 2.5, 7.5 and 15.5 and 8-week-old C57BL male: P0.5 *Scn2a*^fl/fl^/*Emx1*-Cre and *Scn2a*^flfl^ littermates: P9.5 *Scn2a*^fl/fl^/*Vgat*-Cre and *Scn2a*^fl/+^ littermates: P0.5 *Scn2a*^KO/KO^ and *Scn2a*^+/+^ littermates: both sex, C57BL/6J and 129 mixed background) were deeply anesthetized and perfused transcardially with periodate-lysine-4% paraformaldehyde. The brains were removed, and embedded in paraffin. The paraffin-embedded mouse brains were cut in 6 μm sections, deparaffinized, rehydrated, microwaved in 1 mM EDTA, pH 8.0, and blocked in phosphate-buffered saline containing 0.05% Tween 20, 4% BlockAce (Dainippon Sumitomo Pharma, Osaka, Japan) and endogenous avidin and biotin blocker (Vector Laboratories, Burlingame, CA, USA). The sections were then incubated with the goat anti-internal-region Nav1.2 (1:500; SC-31371, G-20, Santa Cruz Biotechnology) and the rabbit anti-Nav1.6 antibody (1:500; II-2)^23^. Endogenous peroxidases were quenched by incubation with 0.3% hydrogen peroxide in phosphate-buffered saline. The sections were further incubated with biotinylated goat polyclonal secondary antibody (1:200; BA-9500, Vector Laboratories). Detection of antibody–antigen complexes was accomplished using the Vectastain Elite ABC kit (PK-6100, Vector Laboratories) and the NovaRed substrate kit (SK-4800, Vector Laboratories).

For immunofluorescence histochemistry, the sections were incubated with the rabbit anti-internal-region Nav1.2 (1:500; ASC-002, Alomone), the goat anti-internal-region Nav1.2 (1:500; SC-31371, G-20, Santa Cruz Biotechnology), the mouse anti-ankyrinG (1:250; SC-12719, Santa Cruz Biotechnology), the goat anti-ankyrinG (1:250; SC-31778, Santa Cruz Biotechnology), the rabbit anti-Tbr1 antibody (1:2,000; ab31940, Abcam), and the rabbit anti-Nav1.6 antibody (10 μg mL^−1^)^23^. Tbr1 is postnatally expressed in the cell nuclei of the majority of glutamatergic neurons in neocortex, except for layer V neurons^65,66^ (Supplementary Fig. 19). The sections were subsequently incubated with the secondary antibodies conjugated with Alexa Flour 488, 594, 647 (1:1,000; Thermo Fisher Scientific) and biotin (1:200; Vector Laboratories). Biotinylated anti-rabbit or anti-goat IgG antibody was detected using the Streptavidin conjugated Alexa Flour 488 or 594 (Thermo Fisher Scientific). Sections were mounted with Antifade Vectashield mounting medium containing 4′-6-diamidino-2-phenylindole (DAPI) (Vector Laboratories) to stain nuclei. Images were captured using Biozero BZ-8100, BZ-X710 microscopes (Keyence, Osaka, Japan) and TCS SP2 microscope (Leica Microsystems, Wetzler, Germany), and processed using Adobe Photoshop Elements 10 (Adobe Systems, San Jose, CA, USA).

### Immuno-electron microscope analysis

Adult C57BL mice at 2–3 months of age were used. The detailed procedure was described previously^67^. Briefly, mice were anesthetized with mixed anesthetic agents (medetomidine, midazolam, and butolphanol) and perfused intracardially with 4% paraformaldehyde in 0.1 M sodium phosphate buffer (pH 7.2). The brains were removed, cryoprotected with 30% sucrose in PB, embedded into OCT compound, and sectioned with 18 μm thickness with cryostat. After microwaved, sections were incubated with 10% of normal donkey serum with 0.1% Triton X-100 for 20 min, and stained overnight with primary goat anti-internal-region Nav1.2 antibody (1:300; SC-31371, G-20, Santa Cruz Biotechnology) diluted with 1% bovine serum albumin/0.01% saponin in phosphate-buffered saline, followed by the incubation with 1.4 nm gold conjugated rabbit anti-goat secondary antibody (1:100; Nanogold: Nanoprobes Inc., Yaphank, NY, USA) for 3 h. After 2.5% glutaraldehyde fixation, 1.4 nm gold signals were silver-enhanced with R-Gent SE-EM (Aurion, Wageningen, Netherlands) for 40 min. Stained sections were fixed with 1% OsO4 for 60 min at 4 °C, dehydrated through graded series of ethanol and embedded in Epon. Ultrathin sections (70 nm) were prepared with ultramicrotome (Leica UC7) and stained with uranyl acetate and lead citrate. The sections were observed under a transmission EM (JEOL model 1400 plus: JEOL, Tokyo, Japan).

### Statistical analyses

Statistical analyses were conducted using the Prism 6 statistical package (GraphPad Software, La Jolla, CA, USA) and Excel (Microsoft, Redmond, WA, USA). Comparisons between two genotype groups were performed using Mann–Whitney *U* test and unpaired *t*-test, unless otherwise described. Dependent variables were analyzed using analysis of variance with Tukey’s multiple comparison test. Values with *P* < 0.05 were considered to be significant.

### Data availability

All data supporting the findings of this study are available within the article and its supplementary information file or are available from the corresponding author upon reasonable request.

## Electronic supplementary material

Supplementary Information
