# Supplementary material for: Nav1.2 haplodeficiency in excitatory neurons causes absence-like seizures in mice
Source: Commun Biol. 2018 Jul 19;1:96. doi: 10.1038/s42003-018-0099-2 (PMC6115194; doi:10.1038/s42003-018-0099-2)
Supplement: Supplementary file 1 — Supplementary Information [file 42003_2018_99_MOESM1_ESM.pdf]

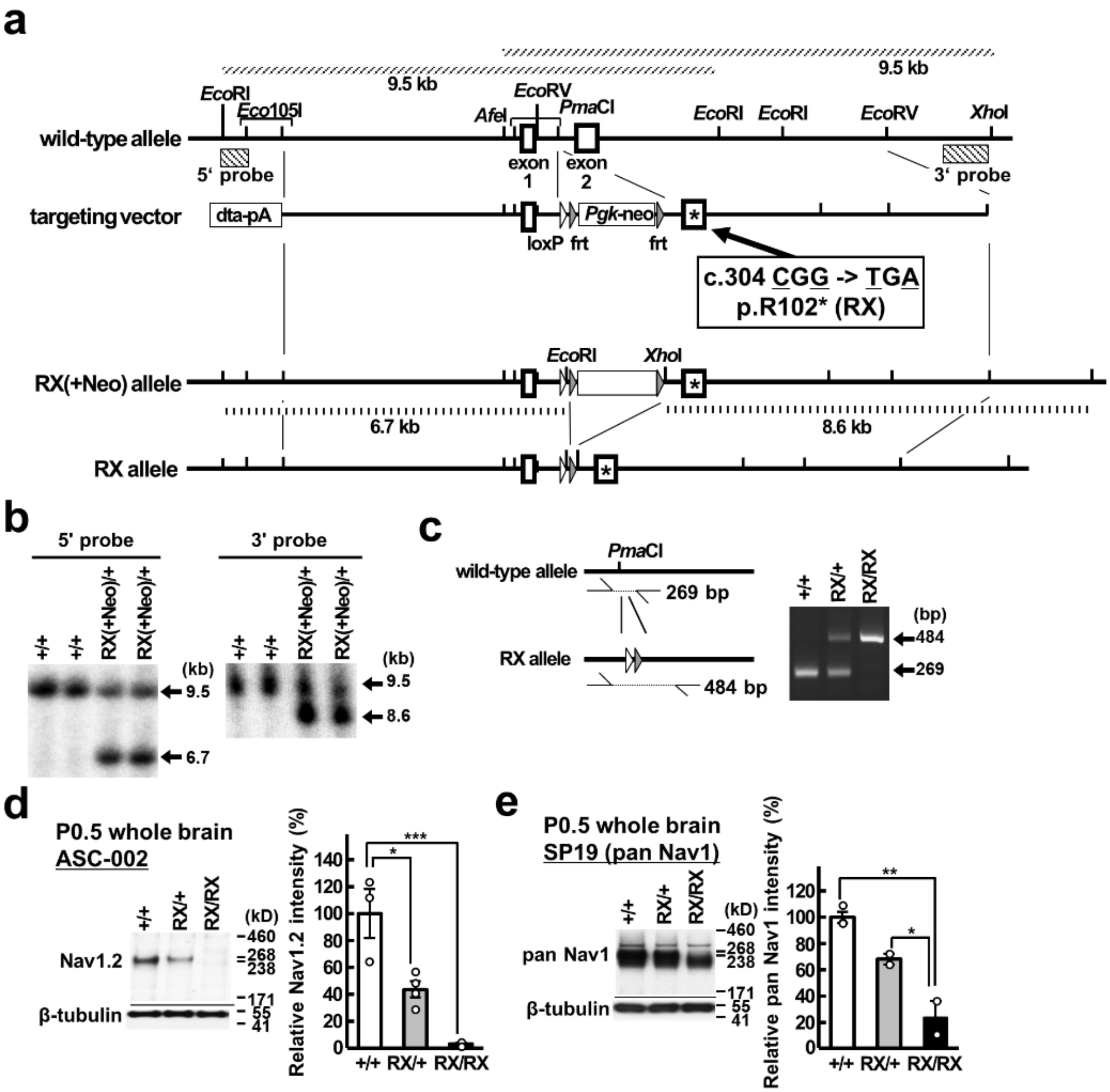

Supplementary Figure 1. Generation of *Scn2a* knock-in mice with R102* mutation. (a) Schematic representation of wild-type allele, targeting vector, RX(+Neo) allele and RX allele. The nucleotide substitution (c.304, CGG to TGA) in exon 2 leading to the nonsense mutation is represented by an asterisk. Restriction enzyme sites and probes used for Southern blot analyses and sizes of restriction fragments detected by the probes are indicated. **(b)** Genotyping of offspring from crosses between male chimeras and C57BL/6J females by Southern blot analyses of *Eco*RI digested or *Afe*I (or its isochizomers, *Eco*47III and *Aor*51HI)-*Xho*I double-digested mouse genomic DNAs. **(c)** Genotyping of offspring from the intercrosses of heterozygotes lacking neomycin cassette by using PCR analyses of their genomic DNAs. Positions of the PCR primers and sizes of PCR products are indicated. **(d, e)** RX allele was effectively inactivated in the *Scn2a*-deficient mice. Western blot analyses of total brain membrane fractions of P0.5 whole brains using anti-Nav1.2 (ASC-002) (**d**) and anti-pan Nav1 (SP19) (**e**) antibodies. Full-length Nav1.2 expression levels were negligible and moderate in $Scn2a^{RX/RX}$ (RX/RX; N = 3) and $Scn2a^{RX/+}$ (RX/+; N = 4) mice, respectively, compared with that in $Scn2a^{+/+}$ (+/+; N = 3) mice [one-way analysis of variance; genotype: F (2, 7) = 20.27, **P = 0.0011, Tukey's test; $Scn2a^{+/+}$ *vs*. $Scn2a^{RX/+}$, *P = 0.0130: $Scn2a^{+/+}$ *vs*. $Scn2a^{RX/RX}$, ***P = 0.0009: $Scn2a^{RX/+}$ *vs*. $Scn2a^{RX/RX}$, P = 0.0551] (**d**). Pan Nav1 immunosignals were apparently reduced in $Scn2a^{RX/RX}$ pups (N = 2), compared to $Scn2a^{+/+}$ (N = 3) mice [one-way analysis of variance; genotype: F (2, 4) = 29.50, **P = 0.0040, Tukey's test; $Scn2a^{+/+}$ *vs*. $Scn2a^{RX/+}$, P = 0.0689: $Scn2a^{+/+}$ *vs*. $Scn2a^{RX/RX}$, **P = 0.0034: $Scn2a^{RX/+}$ *vs*. $Scn2a^{RX/RX}$, *P = 0.0324] (**e**). β-tubulin was used as an internal control. Data represent means ± SEM, *P < 0.05, **P < 0.01, ***P < 0.001.

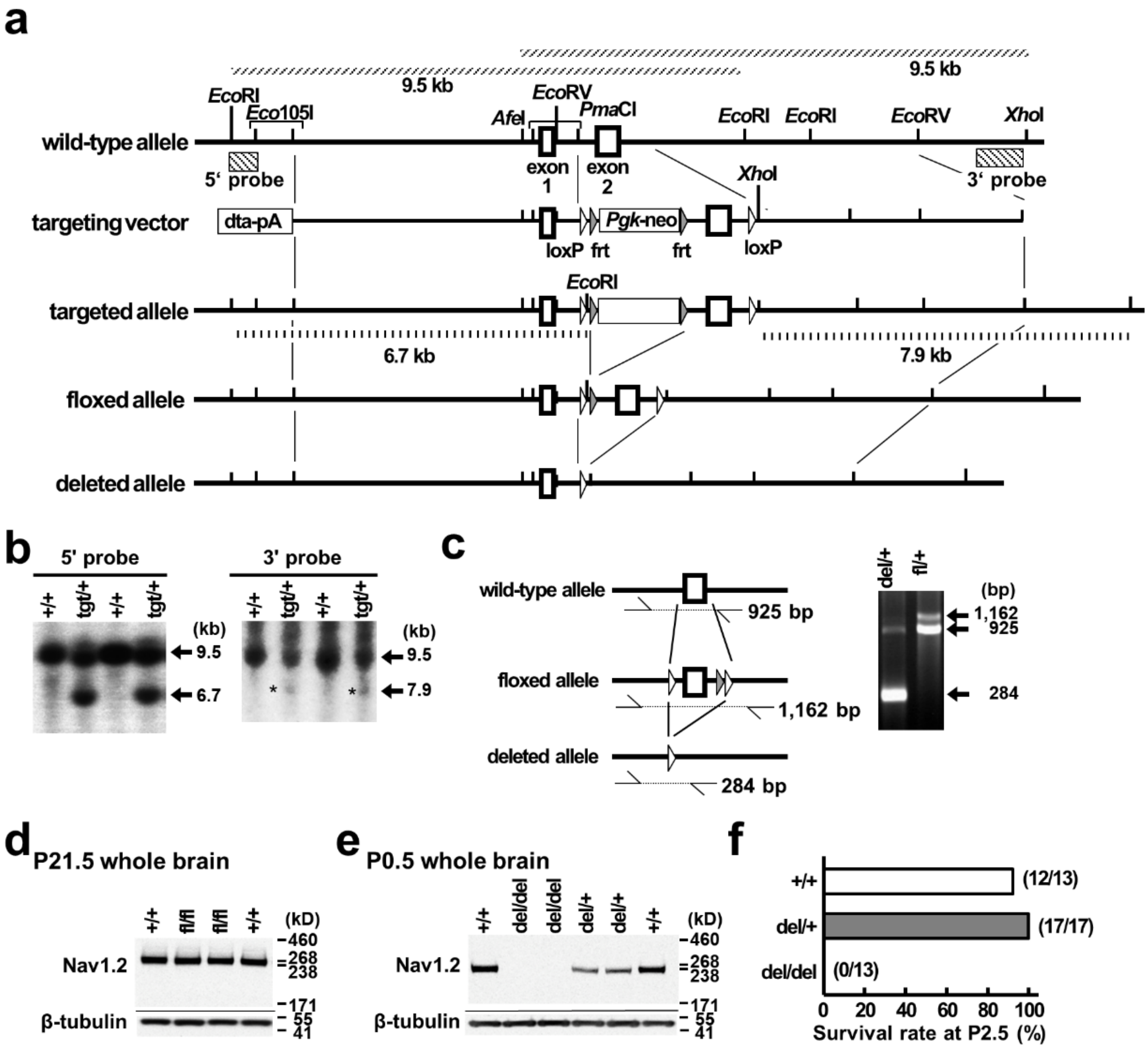

Supplementary Figure 2. Generation of mice with floxed and deleted *Scn2a* alleles. (a) Schematic representation of wild-type *Scn2a* allele, targeting vector, targeted, floxed and deleted alleles. Recognition sites of restriction endonucleases, probes used for Southern blot analyses, and sizes of the restriction fragments detected by the probes are shown. **(b)** Genotyping of offspring from crosses between male chimeras and C57BL/6J females by Southern blot analyses of *Eco*RI digested or *Afe*I (or its isoschizomers, *Eco*47III and *Aor*51HI)-*Xho*I double-digested mouse genomic DNAs. The asterisked 7.9 kb band corresponding to the *Xho*I fragment of the targeted allele (tgt) were barely discernible probably due to DNA methylation at the *Xho*I sites. **(c)** Verification of the floxed (fl) and deleted (del) exon 2 alleles using PCR analyses of genomic DNAs. Primer positions and sizes of PCR products are indicated. **(d)** Western blot analysis of brain membrane proteins prepared from P21.5 *Scn2a*$^{+/+}$ (+/+) and *Scn2a*$^{fl/fl}$ (fl/fl) mice, using anti-Nav1.2 antibody (G-20). **(e)** Western blot analysis of brain membrane proteins prepared from P0.5 wild-type mice, homozygotes or heterozygotes for the deleted *Scn2a* allele, using anti-Nav1.2 antibody (G-20). β-tubulin was used as internal controls (**d, e**). **(f)** Survival rates at P2.5 of *Scn2a*$^{del/del}$ (del/del) (N = 13), *Scn2a*$^{del/+}$ (del/+) (N = 17) and *Scn2a*$^{+/+}$ (+/+) (N = 13) mice. All del/del mice died before P2.5.

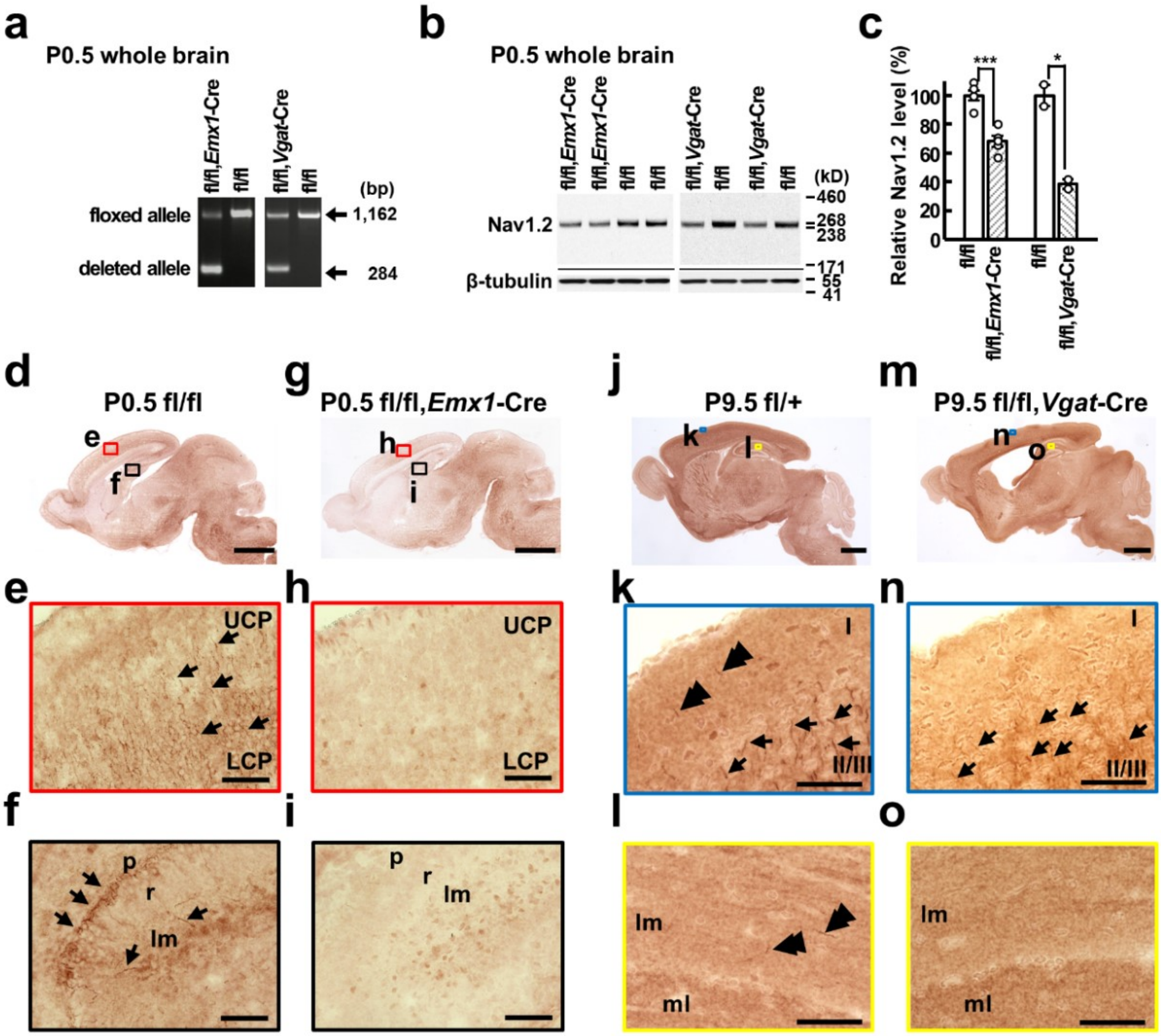

Supplementary Figure 3. Selective *Scn2a* deletion in excitatory or inhibitory neurons. (a) PCR analysis of whole brain genomic DNA of P0.5 *Scn2a*$^{fl/fl}$/*Emx1*-Cre (fl/fl,*Emx1*-Cre), *Scn2a*$^{fl/fl}$/*Vgat*-Cre (fl/fl,*Vgat*-Cre), and control *Scn2a*$^{fl/fl}$ (fl/fl) mice. Cre-loxP-mediated *Scn2a* deletions occurred in an *Emx1*-Cre or *Vgat*-Cre dependent manner. **(b, c)** Western blot analyses of P0.5 whole brains for *Scn2a*$^{fl/fl}$/*Emx1*-Cre, *Scn2a*$^{fl/fl}$ control littermates (N = 5, each genotype), *Scn2a*$^{fl/fl}$/*Vgat*-Cre and *Scn2a*$^{fl/fl}$ control mice (N = 2, each genotype). Unpaired t-test, *Scn2a*$^{fl/fl}$ versus *Scn2a*$^{fl/fl}$/*Emx1*-Cre: t(8) = 6.196, ***P = 0.0003; *Scn2a*$^{fl/fl}$ versus *Scn2a*$^{fl/fl}$/*Vgat*-Cre: t(2) = 7.569, *P = 0.017. **(d-o)** Nav1.2 immunosignals are significantly reduced in cortical and hippocampal excitatory neurons in *Scn2a*$^{fl/fl}$/*Emx1*-Cre mice, and in cortical and hippocampal inhibitory neurons in *Scn2a*$^{fl/fl}$/*Vgat*-Cre mice. Representative parasagittal sections of P0.5 *Scn2a*$^{fl/fl}$ (fl/fl) (**d**), P0.5 *Scn2a*$^{fl/fl}$/*Emx1*-Cre (**g**), P9.5 *Scn2a*$^{fl/+}$ (**j**) and P9.5 *Scn2a*$^{fl/fl}$/*Vgat*-Cre (**m**) brains, stained with anti-Nav1.2 antibody (G-20). Higher magnification images outlined in (**d, g, j, m**) are shown in (**e, f, h, i, k, l, n, o**). Arrows indicate Nav1.2-immunoreactive proximal neurites, which putatively correspond to AISs of excitatory neurons. Doublearrowheads indicate Nav1.2-immunoreactive proximal neurites, which putatively correspond to AISs of inhibitory neurons. Scale bars: (**d, g, j, m**) 1 mm; (**e, f, h, i**) 100 µm; (**k, l, n, o**) 50 µm. UCP, upper cortical plate; LCP, lower cortical plate; o, stratum oriens; p, stratum pyramidale; r, stratum radiatum; lm, stratum lucidum; ml, molecular layer of dentate gyrus. The brain slices were processed in parallel. Shown are representative images of four or more slices per genotype. Data represent means ± SEM, *P < 0.05, ***P < 0.001.

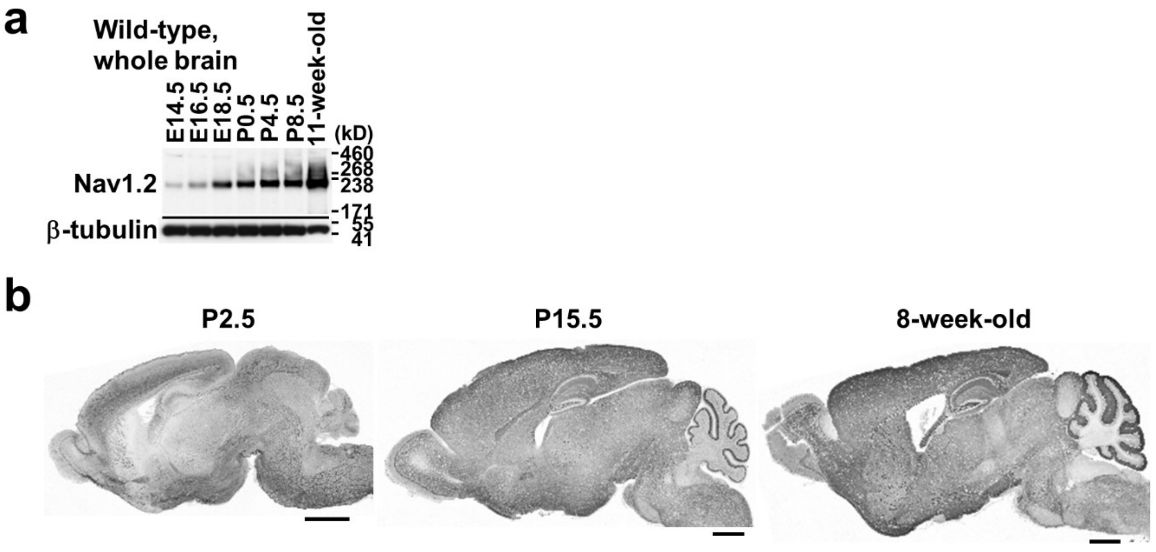

Supplementary Figure 4. Developmental increase in Nav1.2 expression in mouse brain. (a) Western blot of membrane fractions from whole brains at multiple developmental stages probed with anti-Nav1.2 antibody (ASC-002). Anti-β-tubulin antibody was used as internal control. **(b)** Immunohistochemical detection of Nav1.2 in P2.5 (left), P15.5 (middle) and 8-week-old (right) wild-type brains with anti-Nav1.2 antibody (G-20). The brain slices were processed in parallel. Shown are representative images of four or more slices per stage. Scale bars: 1mm.

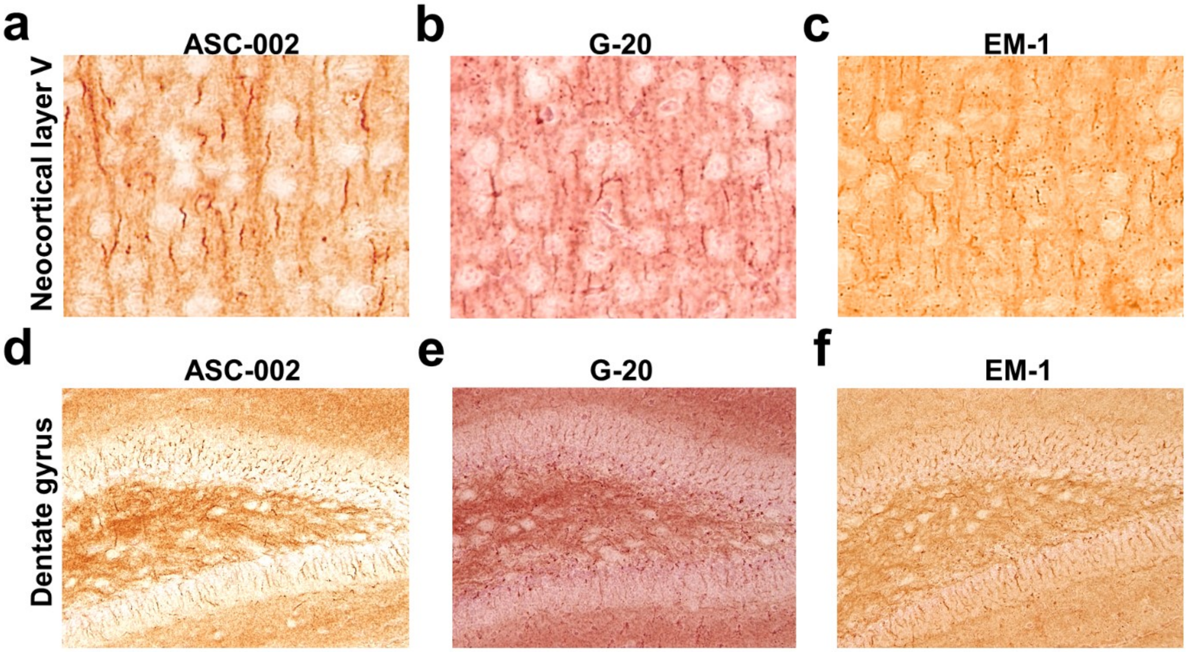

Supplementary Figure 5. Immunohistological patterns of anti-Nav1.2 antibodies. Neocortical layer V (**a-c**) and hippocampal dentate gyrus (**d-f**) of P15.5 wild-type mouse brains were probed with the three anti-Nav1.2 antibodies recognizing distinct epitopes. See Fig.1a for the locations of epitopes for antibodies ASC-002 (**a**, **d**) and EM-1 (**c**, **f**). The epitope for G-20 (**b**, **e**) has not been disclosed. All antibodies showed very similar patterns of immunoreactive signals.

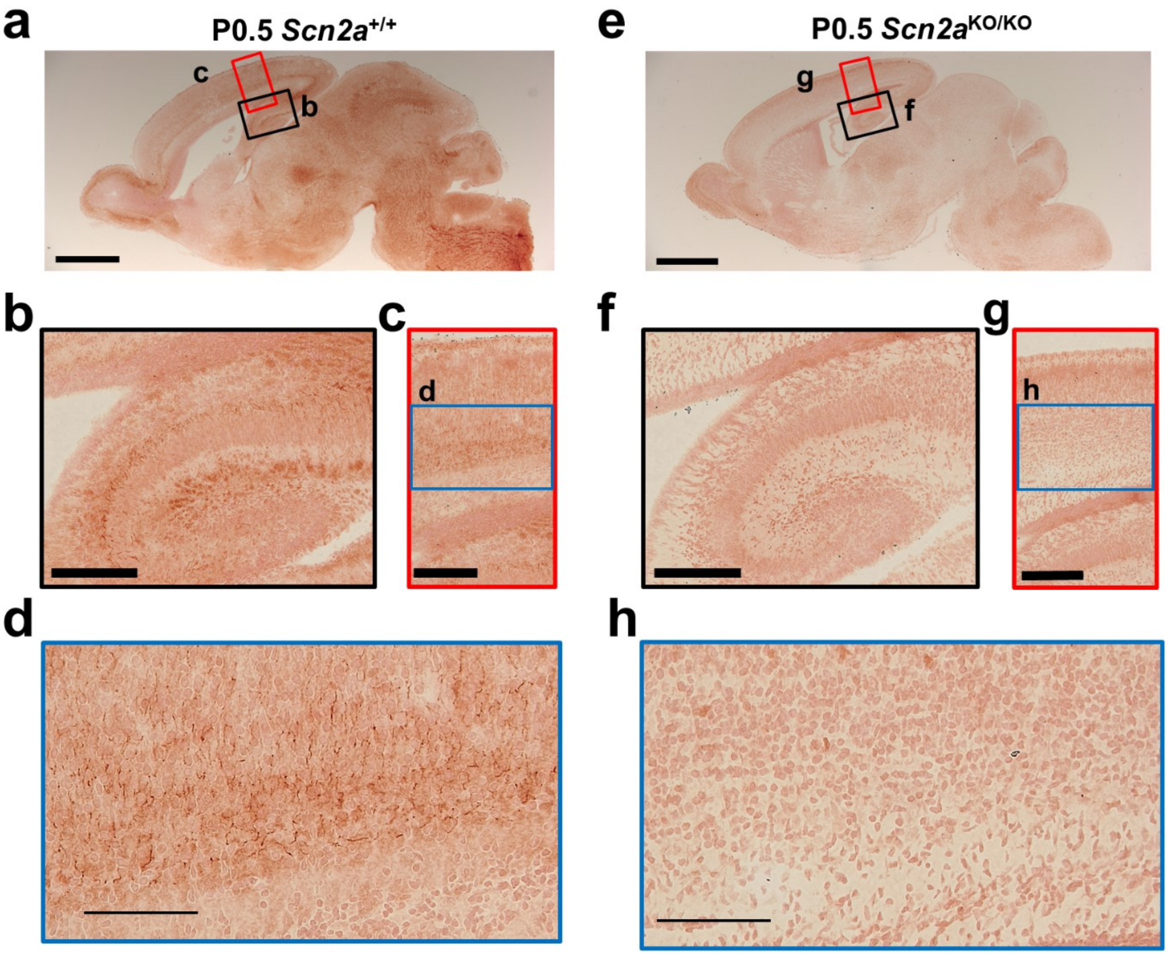

Supplementary Figure 6. Disappeared Nav1.2 immunoreactivity in *Scn2a*$^{KO/KO}$ brain. Brain sections of P0.5 *Scn2a*$^{+/+}$ (**a-d**) and *Scn2a*$^{KO/KO}$ (**e-h**) were stained by anti-Nav1.2 antibody (G-20). Whole brain images (**a, e**), and hippocampus (**b, f**) and neocortex (**c, d, g, h**) are shown. Images outlined in (**a, c, e, g**) are magnified in (**b-d**, **f-h**). Note that the fiber-like dense Nav1.2 signals in *Scn2a*$^{+/+}$ (**a-d**) well disappeared in *Scn2a*$^{KO/KO}$ (**e-h**). The brain slices were processed in parallel. Shown are representative images of four or more slices per genotype. Scale bars, (**a, e**) 1 mm; (**b**, **c**, **f**, **g**) 50µm; (**d**, **h**) 20 µm.

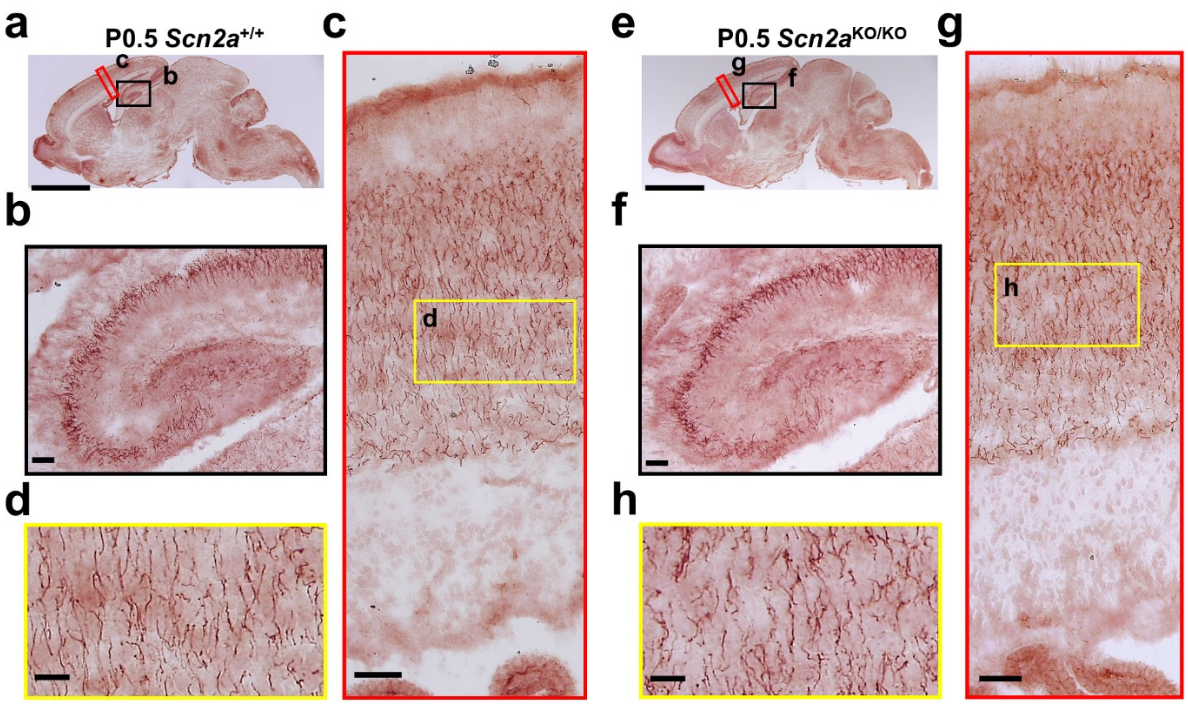

Supplementary Figure 7. No structural alterations in *Scn2a*$^{KO/KO}$ brain. Brain sections of P0.5 *Scn2a*$^{+/+}$ (**a-d**) and *Scn2a*$^{KO/KO}$ (**e-h**) were stained by anti-ankyrin G antibody. Whole brain images (**a, e**), hippocampus (**b, f**) and neocortex (**c, d, g, h**) are shown. Higher-magnification images outlined in (**a, c, e, g**) are shown in (**b-d, f-h**). No significant differences were observed between *Scn2a*$^{+/+}$ (**a-d**) and *Scn2a*$^{KO/KO}$ (**e-h**) for Ankyrin G-positive neurites. The brain slices were processed in parallel. Shown are representative images of four or more slices per genotype. Scale bars, (**a**, **e**) 1 mm; (**b**, **c**, **f**, **g**) 50µm; (**d**,**h**) 20µm.

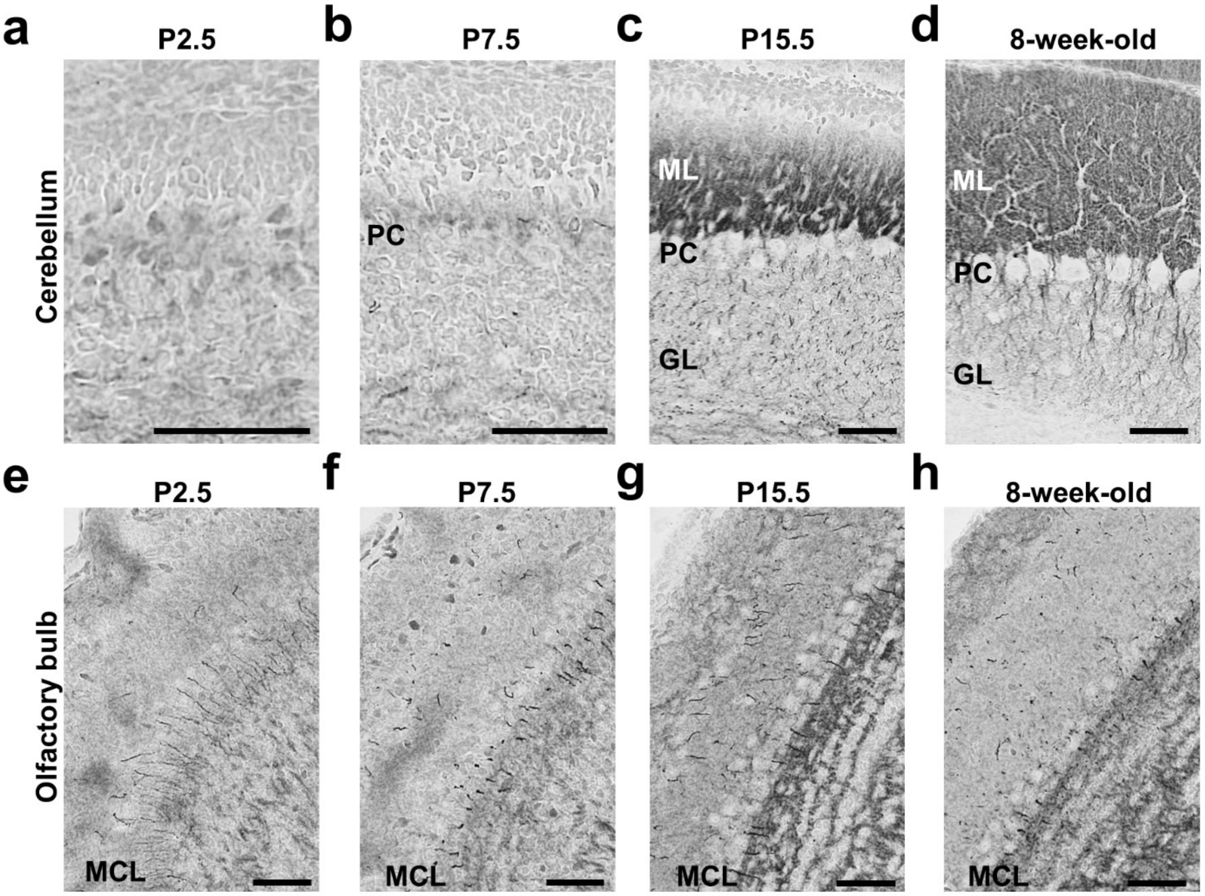

Supplementary Figure 8. Developmental changes of Nav1.2 subcellular distributions. Immunohistochemistry of cerebellum (**a-d**) and olfactory bulb (**e-h**) in P2.5 (**a, e**), P7.5 (**b, f**), P15.5 (**c, g**) or 8-week-old (**d, h**) wild-type mouse brains stained with anti-Nav1.2 antibody (G-20). Note that Nav1.2 immunosignals become highly dense in cerebellar parallel fibers (**c, d**) and show significant changes of distributions in olfactory bulb (**e-h**). GL, granule cell layer; PC, Purkinje cell; ML, molecular layer; MCL, mitral cell layer. The brain slices were processed in parallel. Shown are representative images of four or more slices per stage. Scale bars, 50 µm.

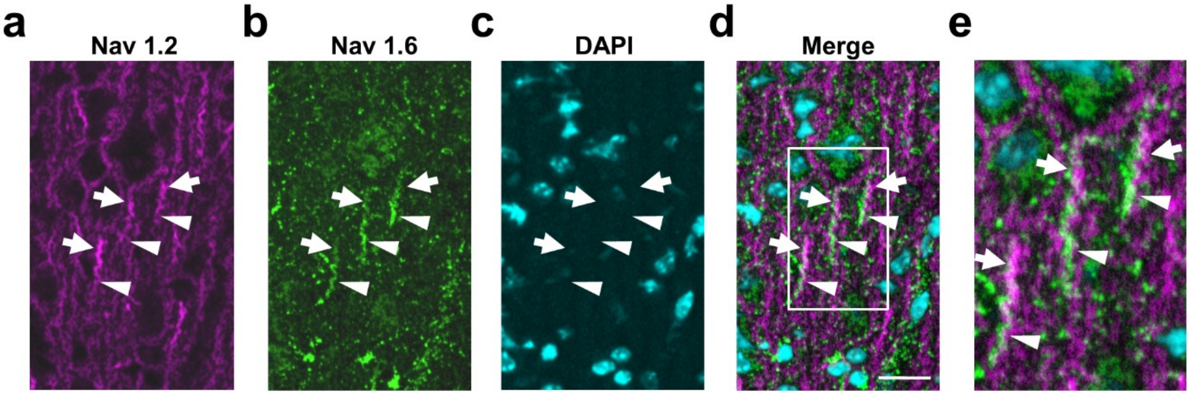

Supplementary Figure 9. Expressions of Nav1.2 and Nav1.6 at the AISs of pyramidal neurons. Immunofluorescence histochemistry of P15.5 wild-type neocortices stained with anti-Nav1.2 (G-20) (**a**; red) and anti-Nav1.6 (**b**; green) antibodies and counter-stained with DAPI (**c**; blue). Merged image (**d**). Higher-magnified image outlined in (**d**) is shown in (**e**). Note that Nav1.2 and Nav1.6 are localized at proximal (arrows) and distal (arrowheads) regions of AISs of pyramidal neurons, respectively. Shown are representative images. Scale bars, 20 μm.

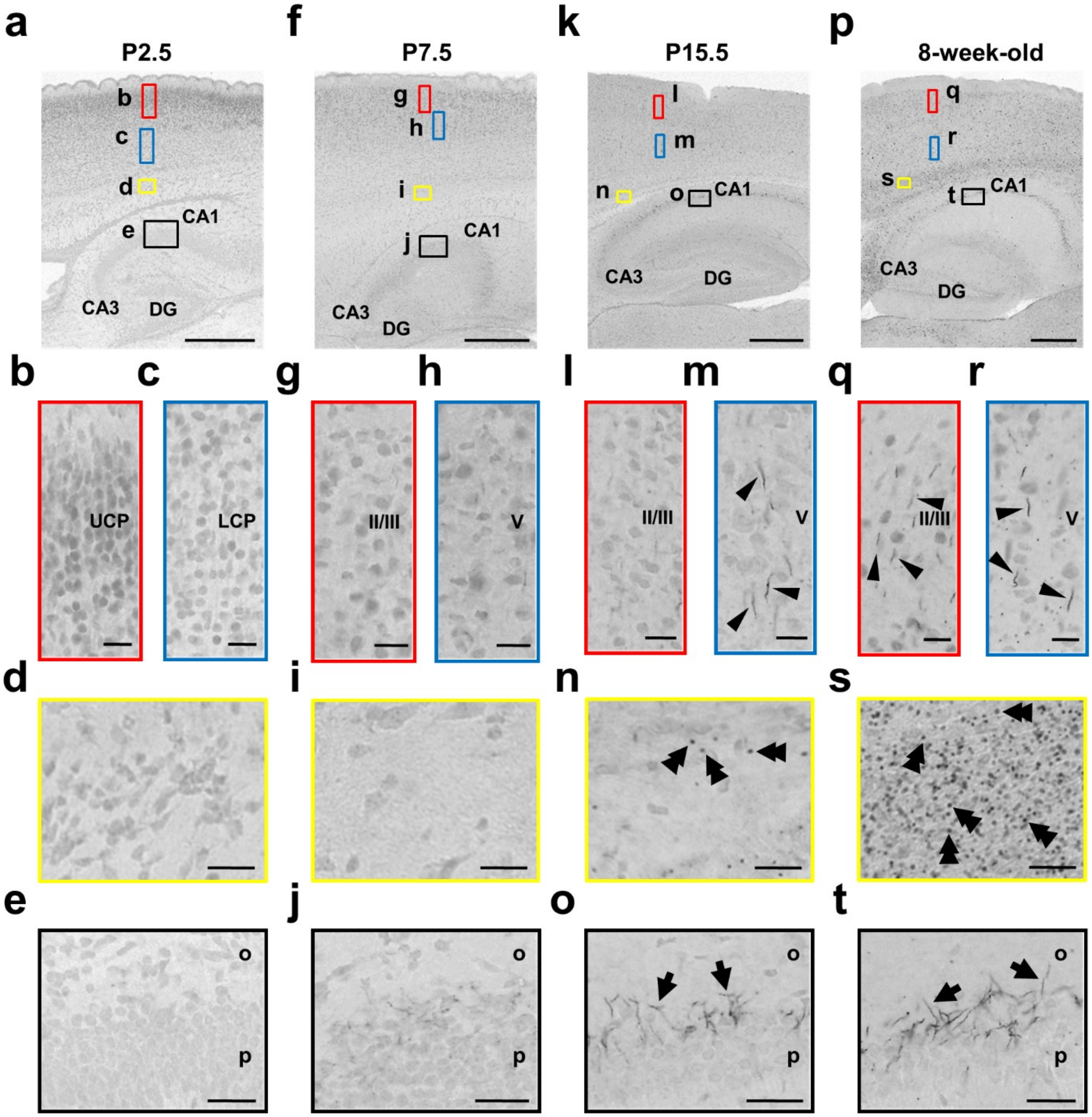

Supplementary Figure 10. Developmental changes of Nav1.6 subcellular distribution. Developmental changes of Nav1.6 histological distribution in neocortex and hippocampus. Brain sections of mice at P2.5 (**a-e**), P7.5 (**f-j**), P15.5 (**k-o**) and 8-week-old (**p-t**) were stained by anti-Nav1.6 antibody. Higher-magnified images outlined in (**a, f, k, p**) are shown in (**b-e, g-j, l-o, q-t**). Note that Nav1.6 at AISs of neocortical pyramidal cells (arrowheads), nodes of Ranvier (double arrowheads) in white matter and AISs of hippocampal pyramidal cells (arrows) continued to be increased until 8-week-old of age. In contrast to the diffuse Nav1.2 signals constantly increased in neocortical layers (Fig. 5c-l), Nav1.6 did not show such diffuse signals in the corresponding regions (**a-r**). DG, dentate gyrus; UCP, upper cortical plate; LCP, lower cortical plate; o, stratum oriens; p, stratum pyramidale. The brain slices were processed in parallel. Shown are representative images of four or more slices per stage. Scale bars: (**a, f, k, p**) 200 µm; (**b, c, g, h, l, m, q, r**) 10 µm; (**d, e, i, j, n, o, s, t**) 20 µm.

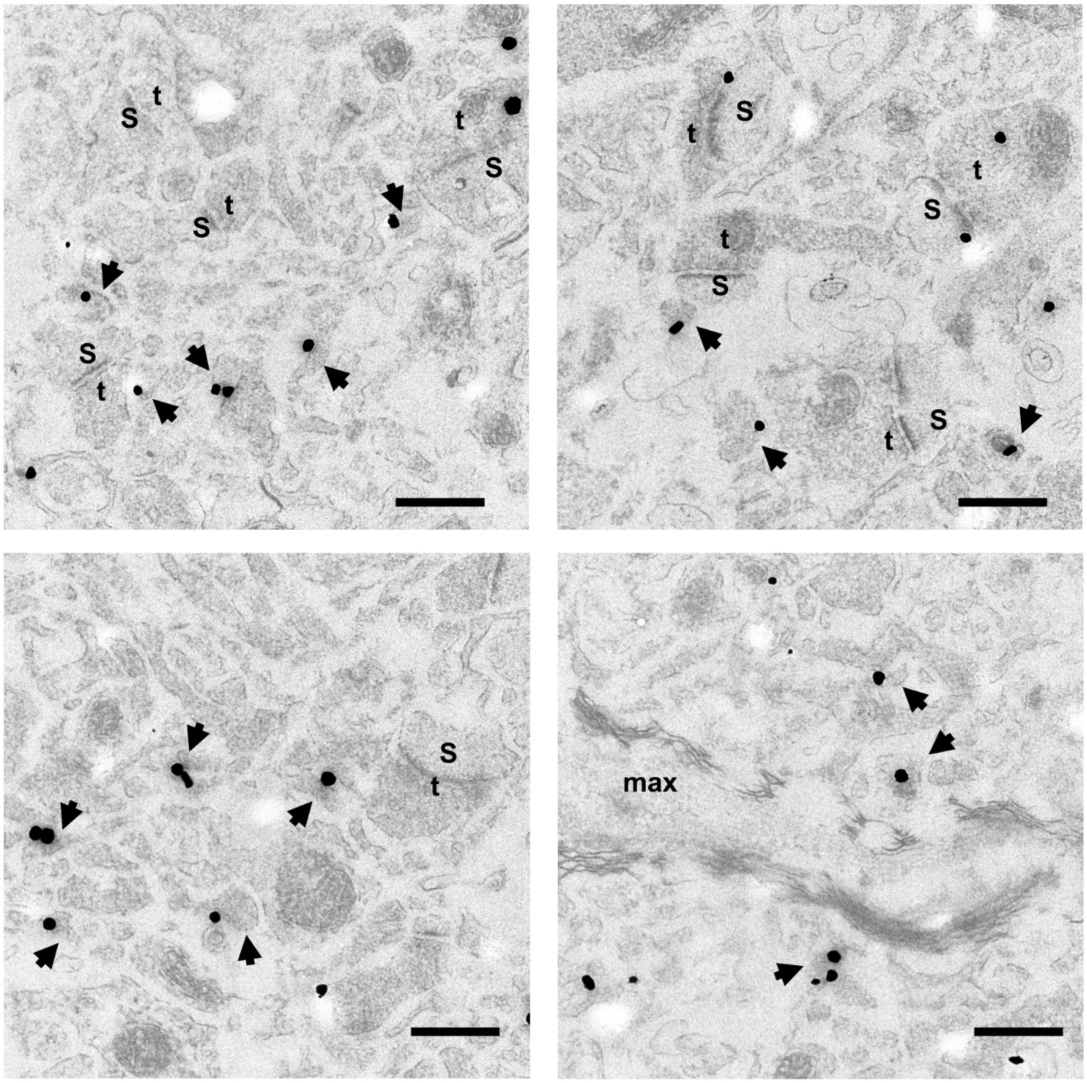

Supplementary Figure 11. Distribution of Nav1.2 in layer II/III of cerebral cortex. Metal particles for Nav1.2 were frequently detected on the cell membrane of some thin process as non-myelinated nerve fibers (arrows), and rarely in myelinated segments of axons (max), synaptic structures as axon terminals (t) or dendritic spines (s). Shown are representative images. Scale bars: 500 nm.

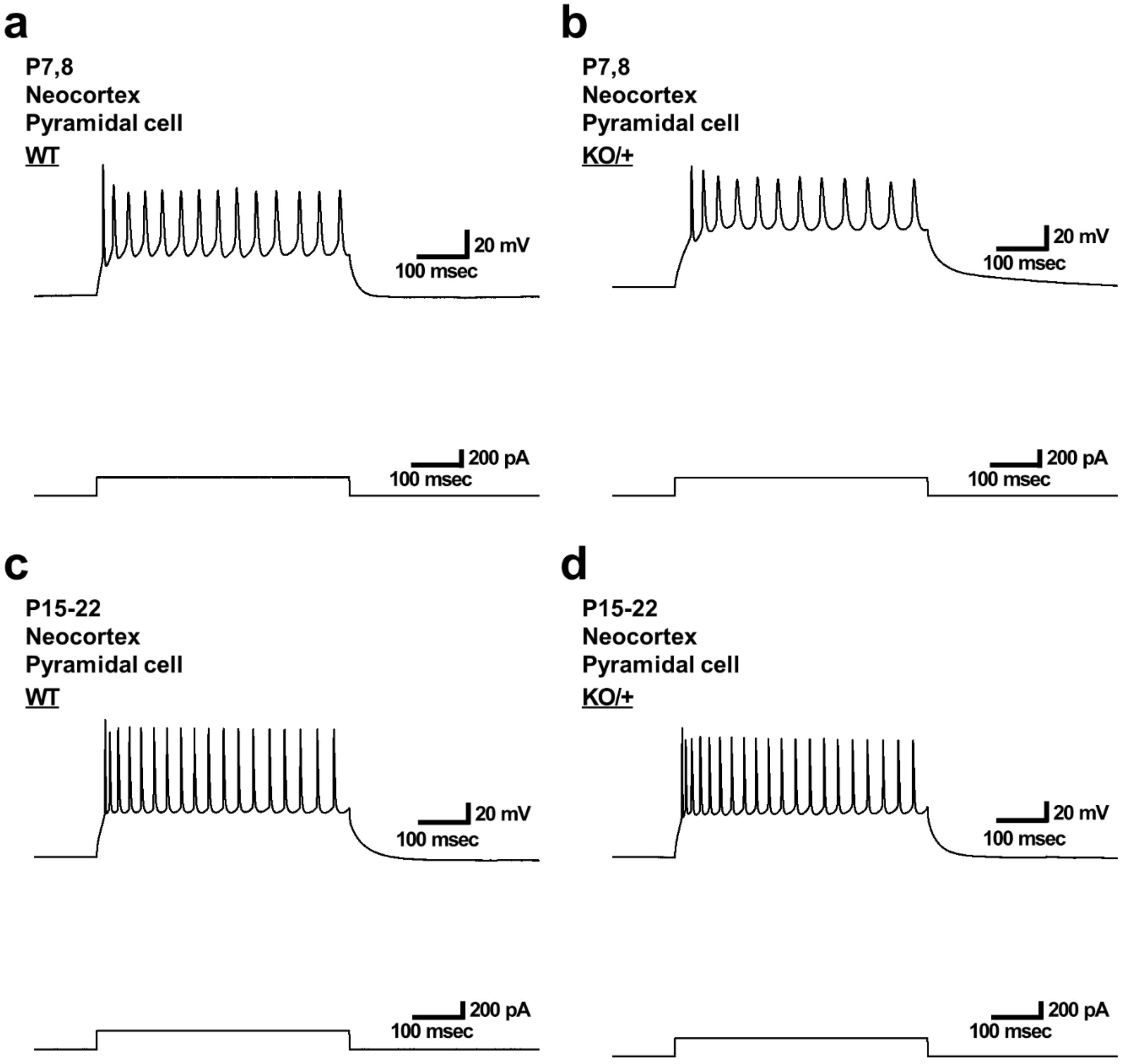

Supplementary Figure 12. Action potentials of *Scn2a*$^{KO/+}$ excitatory neurons. Representative traces of P7-8 (**a, b**) and P15-22 (**c, d**) neocortical pyramidal excitatory neurons from *Vgat*-Venus/*Scn2a*$^{+/+}$ mice (WT) (**a, c**) and *Vgat*-Venus/*Scn2a*$^{KO/+}$ mice (KO/+) (**b, d**) to a depolarizing current pulse at 5 X threshold for 500 msec (WT: n = 19 neurons at P7,8, n = 22 neurons at P15-22, KO/+: n = 16 neurons at P7-8, n = 22 neurons at P15-22).

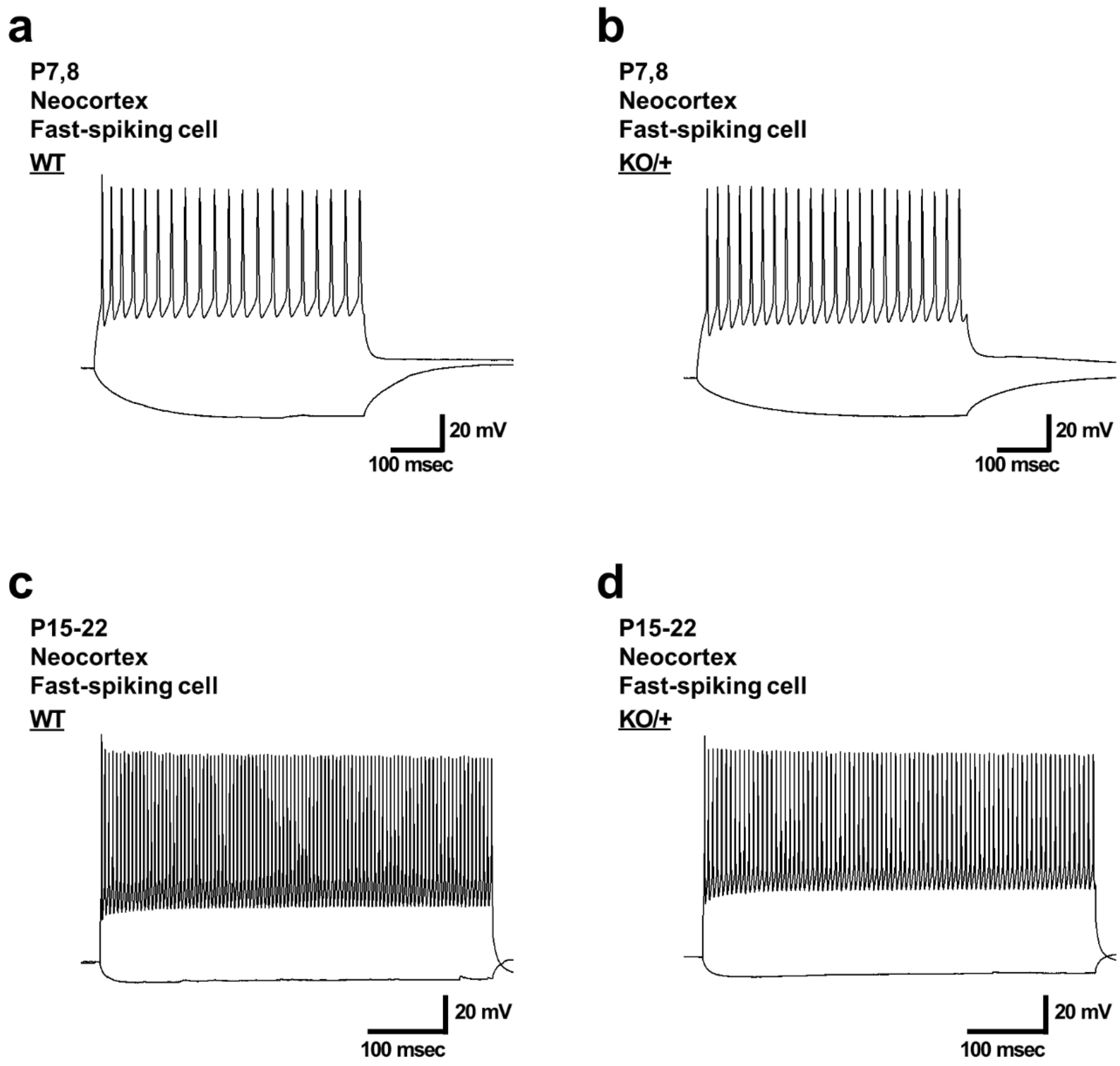

Supplementary Figure 13. Action potentials of *Scn2a*$^{KO/+}$ fast-spiking neurons. Representative traces of P7-8 (**a, b**) and P15-22 (**c, d**) neocortical fast-spiking neurons from *Vgat*-Venus/*Scn2a*$^{+/+}$ mice (WT) (**a, c**) and *Vgat*-Venus/*Scn2a*$^{KO/+}$ mice (KO/+) (**b, d**) to a depolarizing current pulse at 5 X threshold for 500 msec (WT: n = 8 neurons at P7,8, n = 11 neurons at P15-22, KO/+: n = 11 neurons at P7-8, n = 13 neurons at P15-22).

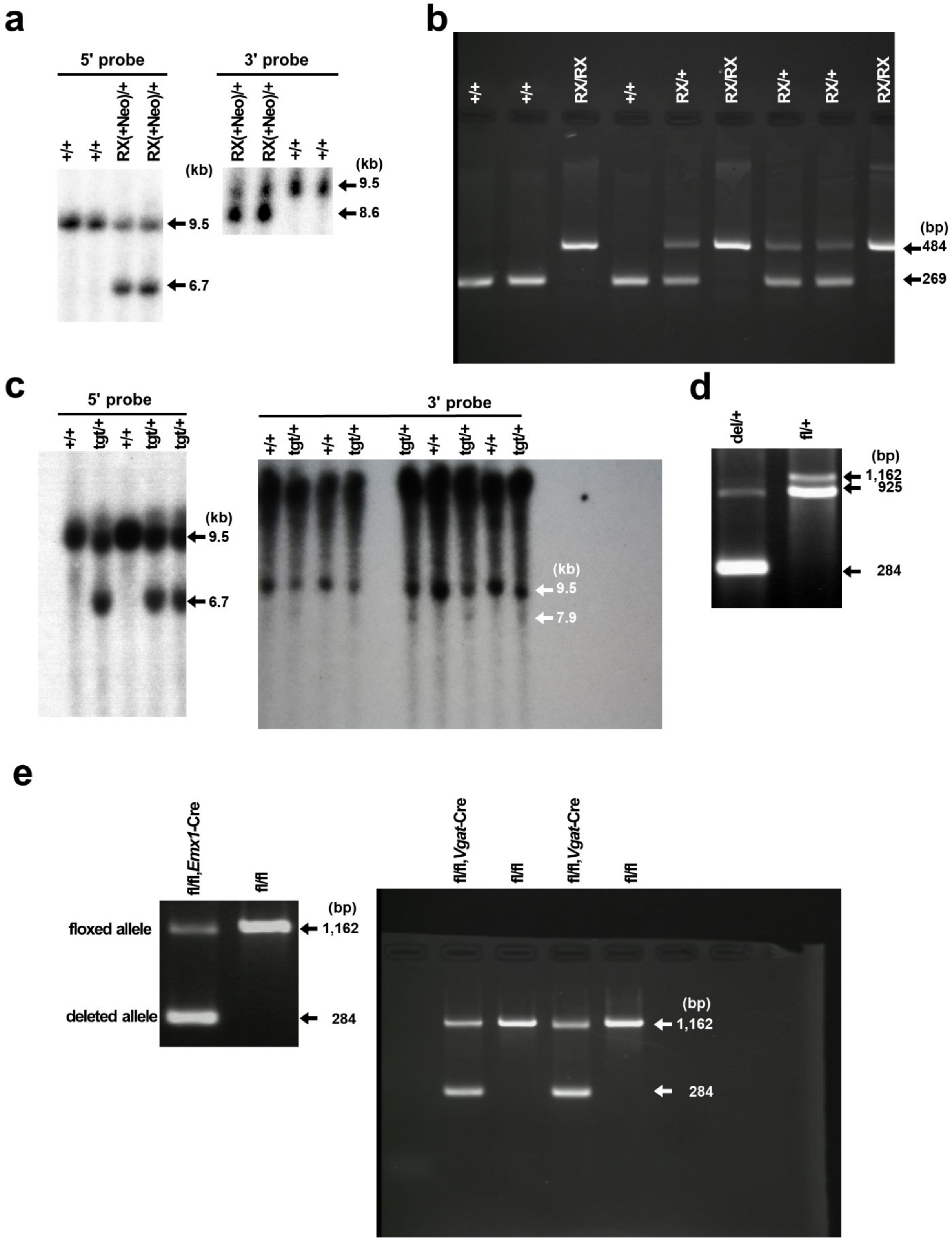

Supplementary Figure 14. Original images of genotyping Southern blots and gels after PCR correspond to Supplementary Figs. 1b (a), 1c (b), 2b (c), 2c (d), and 3a (e).

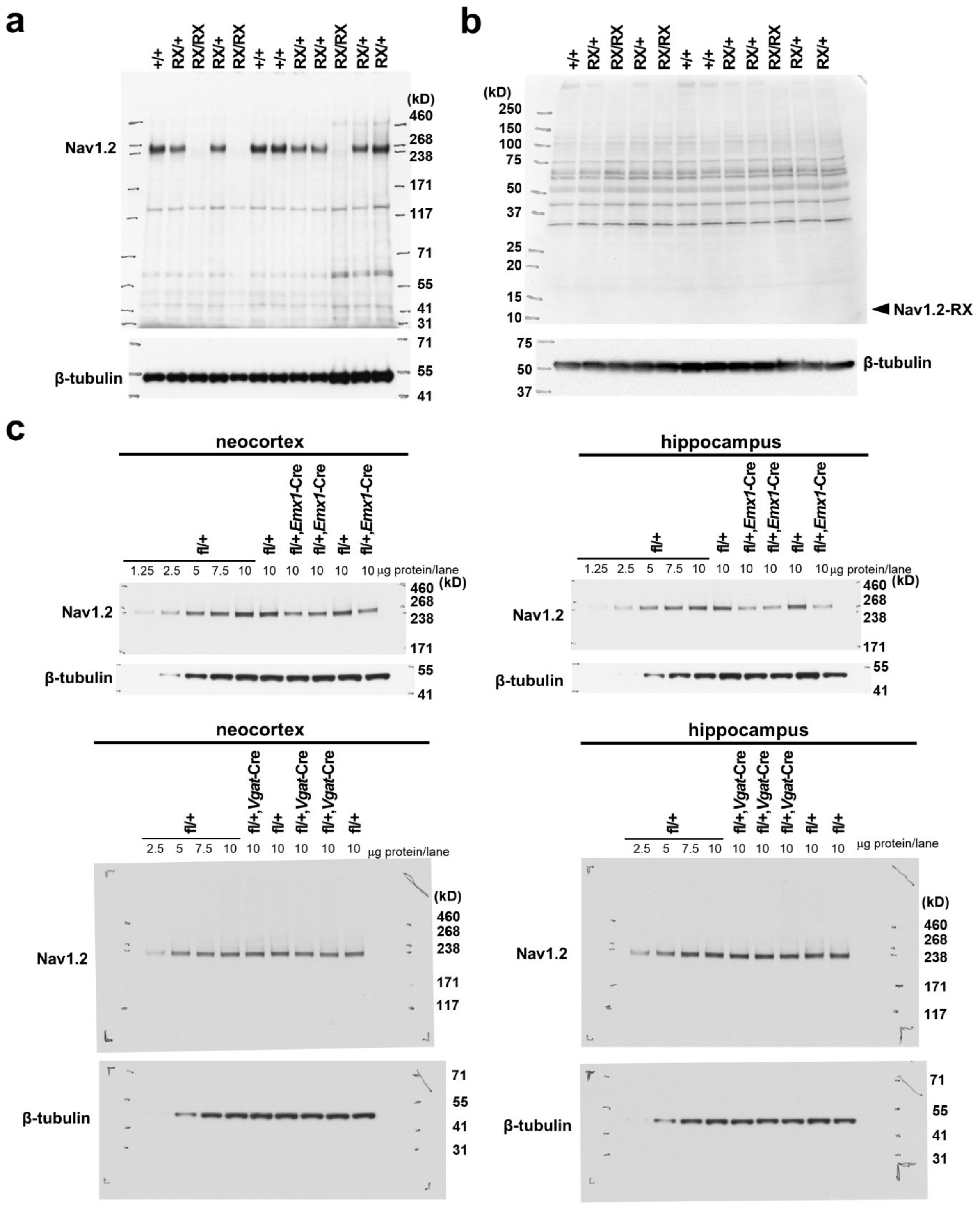

Supplementary Figure 15. Unprocessed original scan images of Western blot films correspond to Figs. 1b (a), 1c (b), and 6 (c).

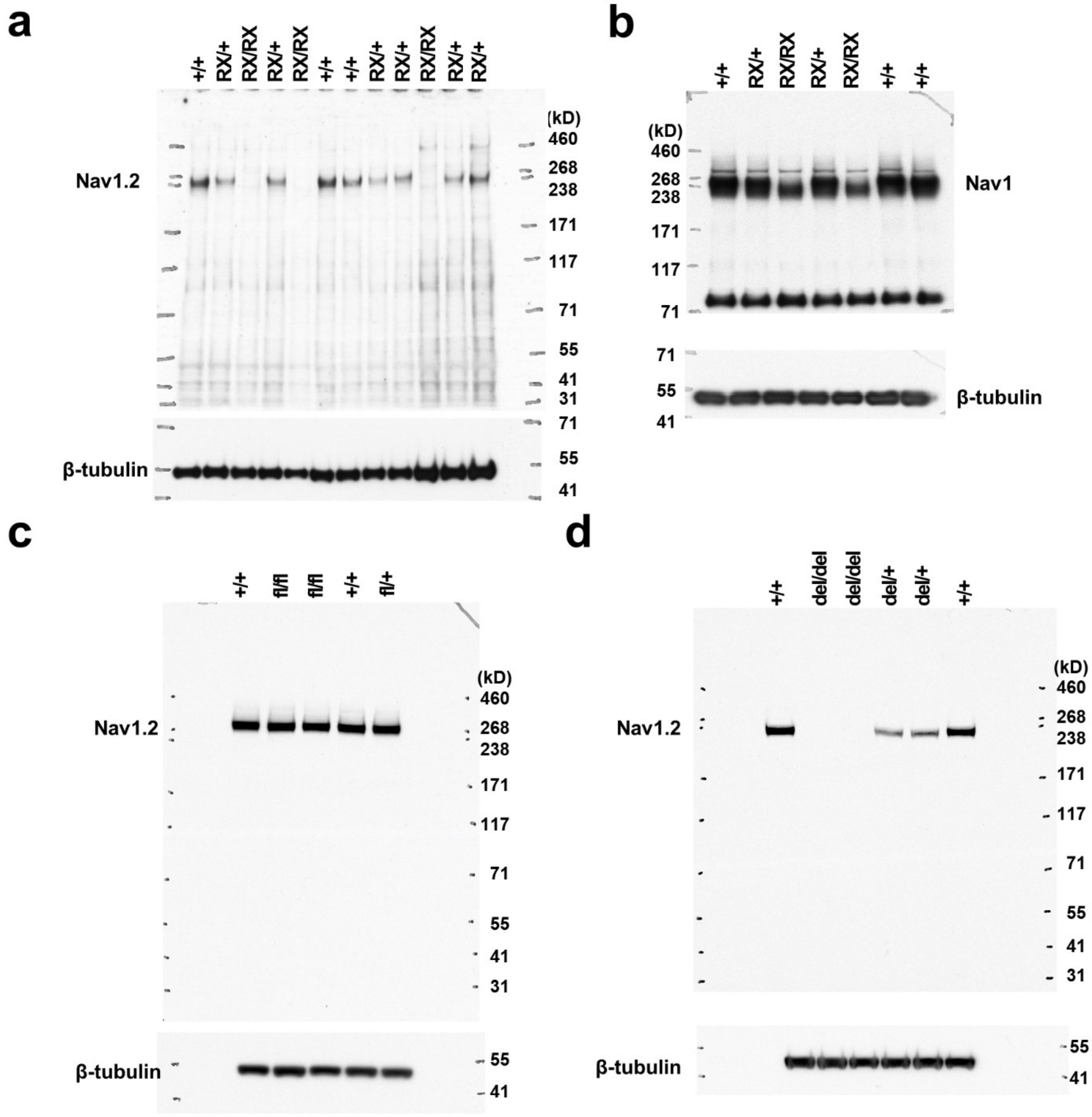

Supplementary Figure 16. Unprocessed original scan images of Western blot films correspond to Supplementary Figs. 1d (a), 1e (b), 2d (c), and 2e (d).

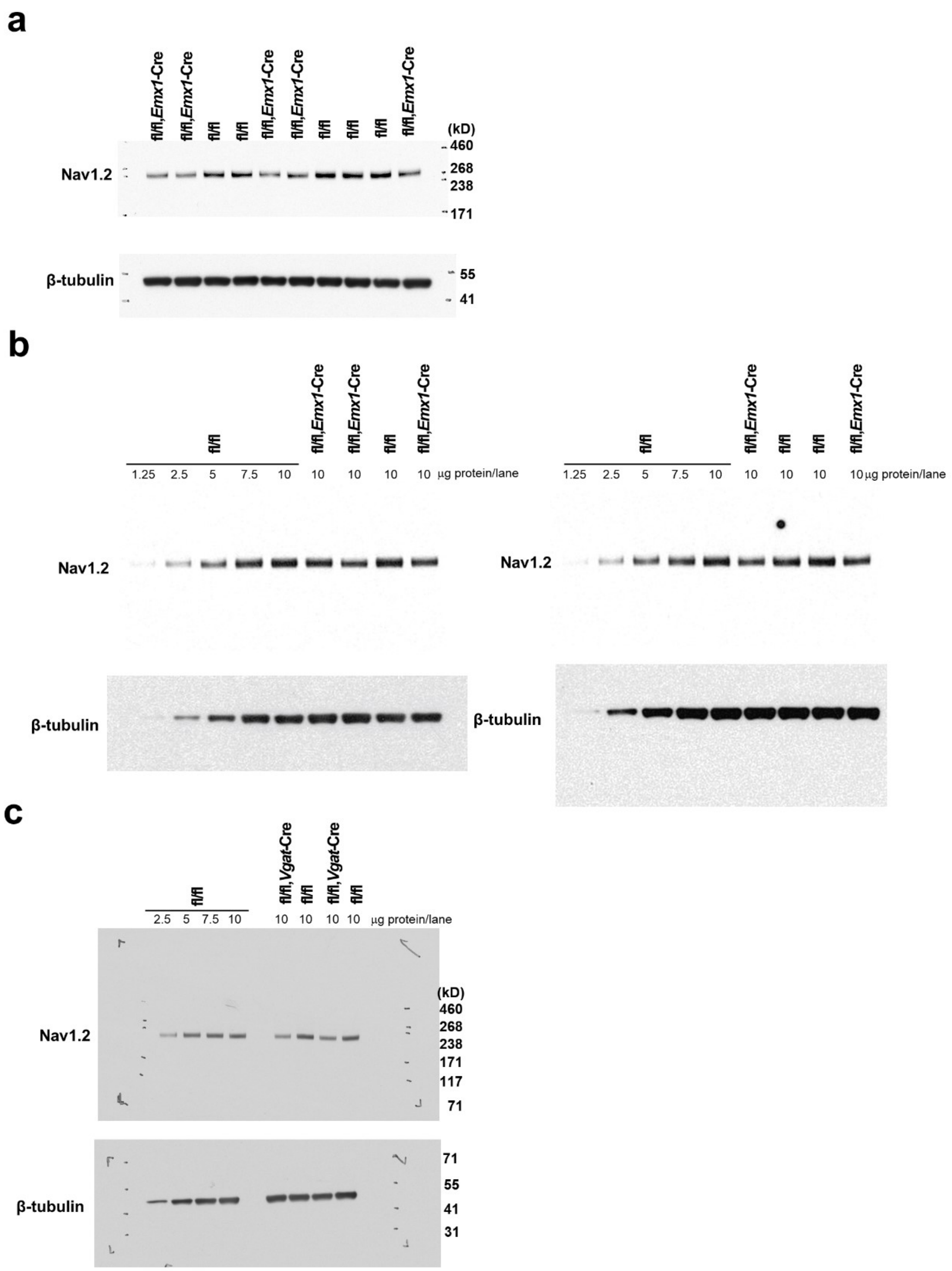

Supplementary Figure 17. Unprocessed original scan images of Western blot films correspond to Supplementary Figs. 3b. Unprocessed original scan images of Nav1.2 expression in *Scn2a*$^{fl/fl}$/*Emx1*-Cre and *Scn2a*$^{fl/fl}$ mice for presentation (**a**) and quantification (**b**), and those in *Scn2a*$^{fl/fl}$/*Vgat*-Cre and *Scn2a*$^{fl/fl}$ mice (**c**). The blots in (**b**) were processed in parallel.

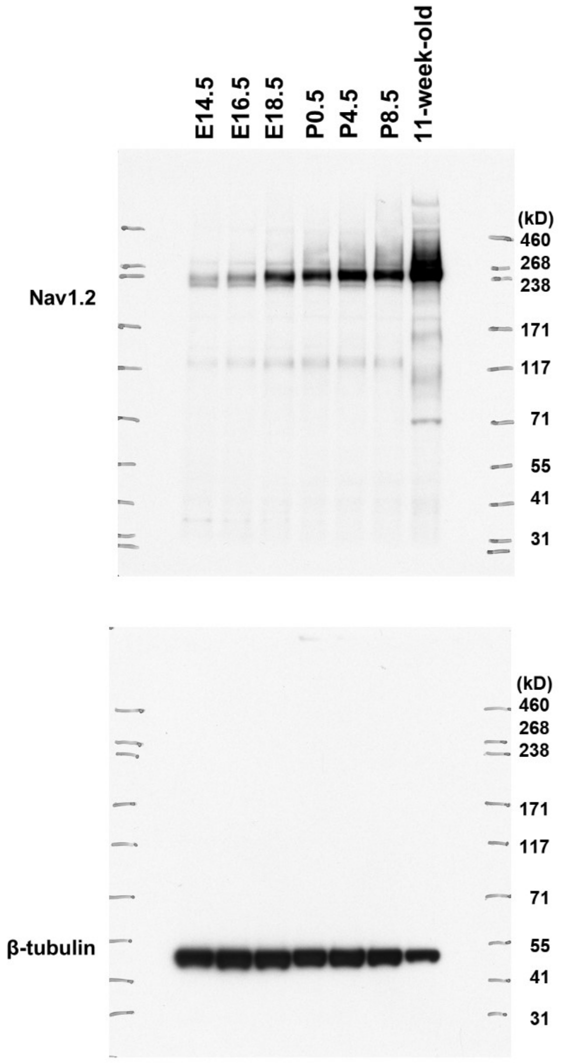

Supplementary Figure 18. Unprocessed original scan images of Western blot films correspond to Supplementary Fig. 4a.

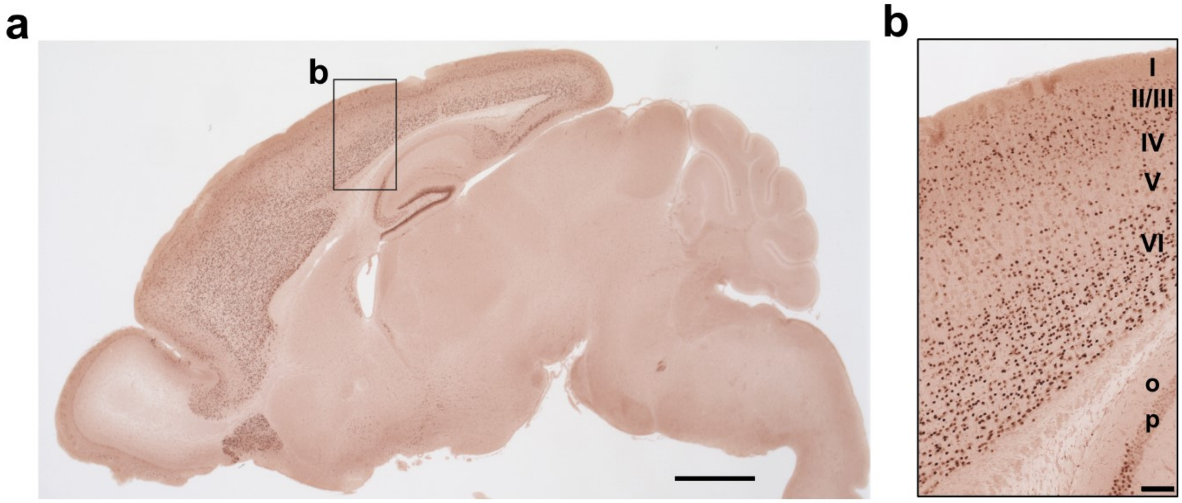

Supplementary Figure 19. Distribution of Tbr1-positive excitatory neurons. Tissues were stained with anti-Tbr1 antibody. A higher-magnified image outlined in (**a**) is shown in (**b**). o, stratum oriens; p, stratum pyramidale. Scale bars: (**a**) 1 mm; (**b**) 100 μm.

Supplementary Table 1. Electrophysiological properties of *Scn2a*$^{KO/+}$ neurons. Single action potentials (APs) and train APs were obtained by 1 x threshold and 5 x threshold current injections, respectively. Decrement of spike firing rate was calculated as the ratio of the last spike number (100 msec) divided by the first spike number (100 msec). In *Vgat*-Venus/*Scn2a*$^{KO/+}$ mice (KO/+), reduction of peak amplitude and broadening of spike width in neocortical pyramidal neurons (P7–8) were significant, and spike width was significantly different at a later stage (P18–22). Spike number in trains did not significantly differ between the genotypes at both age groups. There is no significant difference in fast-spiking interneurons. Cells of *Scn2a*$^{KO/+}$ mice highlighted by blue showed significant differences from *Vgat*-Venus/*Scn2a*$^{+/+}$ mice (WT) (see Supplementary Table 2 for statistical tests). Data represent mean ± SEM., *P < 0.05, **P < 0.01, ***P < 0.001.

Pyramidal neuron		Visual cortex L2/3 pyramidal neuron				Hippocampal CA1 pyramidal neuron			
		P7-8		P18-22		P7-8		P15-20	
		WT	*Scn2a* (KO/+)	WT	*Scn2a* (KO/+)	WT	*Scn2a* (KO/+)	WT	*Scn2a* (KO/+)
	cell number	19	16	22	22	17	22	15	17
	Input resistance (MΩ)	826.7 ± 52.1	805.3 ± 51.3	199.2 ± 10.1	206.1 ± 11.1	454.2 ± 58.4	498.2 ± 30.8	231.0 ± 10.1	227.1 ± 11.9
	Resting membrane potential (mV)	-67.3 ± 1.0	-68.0 ± 1.2	-72.9 ± 1.2	-73.3 ± 1.1	-66.0 ± 1.5	-64.0 ± 1.0	-65.2 ± 0.9	-65.4 ± 0.9
	Injected currents (pA)	51.5 ± 3.4	50.1 ± 3.9	73.7 ± 7.5	86.2 ± 6.0	63.2 ± 5.9	56.3 ± 4.3	50.3 ± 4.1	45.8 ± 4.0
Single-APs	Threshold (mV)	-32.5 ± 0.7	-31.4 ± 0.6	-38.5 ± 1.0	-38.5 ± 0.7	-34.8 ± 0.8	-32.6 ± 0.7	-41.3 ± 0.7	-41.8 ± 1.0
	Peak amplitude (mV)	69.1 ± 1.2	***60.5 ± 0.7	85.7 ± 1.7	85.6 ± 1.2	80.5 ± 1.4	***72.2 ± 1.1	89.7 ± 2.0	89.4 ± 1.5
	Half-width (msec)	2.6 ± 0.1	***3.2 ± 0.1	1.3 ± 0.1	*1.6 ± 0.1	2.2 ± 0.1	2.3 ± 0.1	1.8 ± 0.1	2.1 ± 0.1
Train-APs	1st spike peak amplitude (mV)	68.4 ± 1.3	***59.5 ± 1.1	86.5 ± 2.2	85.5 ± 1.6	79.4 ± 1.2	***69.4 ± 1.5	85.4 ± 4.6	89.9 ± 1.8
	1st spike half-width (msec)	2.1 ± 0.1	***2.6 ± 0.1	1.6 ± 0.0	**1.9 ± 0.1	1.6 ± 0.1	1.6 ± 0.1	1.8 ± 0.1	1.8 ± 0.1
	2nd spike peak amplitude (mV)	48.8 ± 1.7	***39.2 ± 1.6	69.3 ± 2.0	68.2 ± 1.7	69.5 ± 2.2	*63.0 ± 4.2	67.9 ± 4.1	68.1 ± 2.6
	2nd spike half-width (msec)	4.4 ± 0.2	***6.3 ± 0.4	2.2 ± 0.1	**2.7 ± 0.1	2.5 ± 0.3	2.6 ± 0.1	2.5 ± 0.1	2.7 ± 0.1
	Reduction	0.71 ± 0.02	0.65 ± 0.03	0.80 ± 0.02	0.80 ± 0.01	0.87 ± 0.02	0.91 ± 0.01	0.78 ± 0.03	0.76 ± 0.02
	Spike number (/500msec)	11.3 ± 0.7	10.0 ± 0.8	16.0 ± 0.7	16.7 ± 0.8	13.5 ± 0.9	12.9 ± 0.9	15.9 ± 0.8	15.9 ± 0.5

Fast-spiking interneuron		Visual cortex L2/3 fast-spiking interneuron				Hippocampal CA1 fast-spiking interneuron			
		P7-8		P18-22		P7-8		P16-18	
		WT	*Scn2a* (KO/+)	WT	*Scn2a* (KO/+)	WT	*Scn2a* (KO/+)	WT	*Scn2a* (KO/+)
	cell number	8	11	11	13	13	15	9	10
	Input resistance (MΩ)	853.3 ± 74.9	751.9 ± 82.2	123.9 ± 13.4	133.3 ± 9.6	818.4 ± 111.4	811.6 ± 94.6	148.0 ± 17.2	125.8 ± 10.9
	Resting membrane potential (mV)	-66.4 ± 1.4	-65.1 ± 1.9	-67.9 ± 0.8	-67.6 ± 1.0	-64.3 ± 1.1	-63.9 ± 1.1	-63.5 ± 1.3	-62.1 ± 0.8
	Injected currents (pA)	33.8 ± 3.6	41.6 ± 4.3	259.1 ± 22.5	238.5 ± 15.3	71.7 ± 8.0	73.5 ± 6.7	214.6 ± 26.2	229.3 ± 26.8
Single-APs	Threshold (mV)	-34.8 ± 1.7	-35.6 ± 1.3	-35.1 ± 0.8	**-31.1 ± 1.0	-39.6 ± 1.0	**-36.1 ± 0.7	-32.4 ± 2.7	-34.5 ± 1.2
	Peak amplitude (mV)	67.6 ± 1.1	65.8 ± 1.5	68.6 ± 1.8	62.8 ± 2.1	70.9 ± 1.6	67.9 ± 1.7	64.4 ± 4.5	68.8 ± 1.9
	Afterhyperpolarization (mV)	18.6 ± 1.3	18.9 ± 1.3	22.6 ± 1.1	20.9 ± 0.8	18.0 ± 0.8	18.7 ± 0.8	22.0 ± 1.4	18.1 ± 1.3
	Half-width (msec)	1.9 ± 0.1	1.9 ± 0.1	0.5 ± 0.1	0.5 ± 0.1	1.4 ± 0.1	1.4 ± 0.1	0.6 ± 0.1	0.7 ± 0.1
Train-APs	1st spike peak amplitude (mV)	65.0 ± 0.6	63.6 ± 1.6	77.2 ± 1.1	73.5 ± 1.7	68.8 ± 1.7	65.8 ± 1.5	70.0 ± 2.8	73.0 ± 1.8
	1st spike width (msec)	3.0 ± 0.1	3.1 ± 0.2	1.0 ± 0.1	1.0 ± 0.1	1.2 ± 0.1	1.2 ± 0.1	0.6 ± 0.1	0.7 ± 0.1
	2nd spike peak amplitude (mV)	60.5 ± 1.4	56.5 ± 1.9	62.6 ± 1.4	58.9 ± 2.0	66.3 ± 1.9	63.2 ± 1.4	60.1 ± 2.7	59.6 ± 1.6
	2nd spike width (msec)	3.7 ± 0.2	3.9 ± 0.2	1.1 ± 0.1	1.2 ± 0.1	1.4 ± 0.1	1.5 ± 0.1	0.7 ± 0.1	0.7 ± 0.1
	Last spike amplitude (mV)	52.7 ± 2.5	51.1 ± 3.1	50.6 ± 2.2	49.7 ± 1.9	54.6 ± 3.7	53.1 ± 2.8	50.9 ± 2.6	52.2 ± 2.2
	Last spike width (msec)	4.6 ± 0.5	4.7 ± 0.3	1.2 ± 0.1	1.3 ± 0.1	2.7 ± 0.3	2.4 ± 0.2	0.9 ± 0.1	0.8 ± 0.1
	Reduction	0.93 ± 0.02	0.91 ± 0.04	0.81 ± 0.02	0.84 ± 0.01	0.96 ± 0.01	0.96 ± 0.01	0.86 ± 0.02	0.82 ± 0.01
	Spike number (/500msec)	22.5 ± 1.3	23.7 ± 1.6	106.8 ± 4.8	98.4 ± 5.2	26.3 ± 1.0	28.4 ± 0.9	67.3 ± 7.0	70.8 ± 5.8

Supplementary Table 2. Statistical tests for electrophysiological analyses in Supplementary table 1. *Vgat*-Venus/*Scn2a*$^{+/+}$ (WT) and *Vgat*-Venus/*Scn2a*$^{KO/+}$ (KO/+) mice were compared using unpaired t-tests. Cells highlighted by blue showed significant differences between. *P < 0.05, **P < 0.01, ***P < 0.001.

Pyramidal neuron		Visual cortex L2/3 pyramidal neuron		Hippocampal CA1 pyramidal neuron	
		P7-8	P18-22	P7-8	P15-20
	Input resistance	t(33) = 0.2885 P = 0.7748	t(42) = -0.4658 P = 0.6438	t(37) = -1.7615 P = 0.0864	t(30) = 0.2485 P = 0.8054
	Resting membrane potential	t(33) = 0.3482 P = 0.7299	t(42) = 0.2384 P = 0.8128	t(37) = -0.2643 P = 0.7930	t(30) = 0.1439 P = 0.8866
	Injected currents	t(33) = 0.2630 P = 0.7942	t(42) = -1.2948 P = 0.2025	t(37) = 1.0959 P = 0.2802	t(30) = 0.7801 P = 0.4415
Single-APs	Threshold	t(33) = -1.2115 P = 0.2343	t(42) = 0.0161 P = 0.9872	t(37) = -1.5966 P = 0.1189	t(30) = 0.3792 P = 0.7072
	Peak amplitude	t(33) = 6.0638 ***P = 0.0000008	t(42) = 0.0791 P = 0.9373	t(37) = 4.7683 ***P = 0.000029	t(30) = 0.1251 P = 0.9013
	Half-width	t(33) = -4.0342 ***P = 0.00031	t(42) = -3.6442 *P = 0.0109	t(37) = -1.0562 P = 0.2977	t(30) = -1.7730 P = 0.0864
Train-APs	1st spike peak amplitude	t(33) = 5.2324 ***P = 0.000093	t(42) = 0.3736 P = 0.7106	t(37) = 4.6255 ***P = 0.000045	t(30) = -0.9658 P = 0.3419
	1st spike half-width	t(33) = 4.4581 ***P = 0.000090	t(42) = 3.2070 **P = 0.0026	t(37) = -1.0216 P = 0.3136	t(30) = 0.1097 P = 0.9134
	2nd spike peak amplitude	t(33) = 4.0673 ***P = 0.00028	t(42) = 0.4360 P = 0.6651	t(37) = 2.3993 *P = 0.0216	t(30) = -0.0494 P = 0.9609
	2nd spike half-width	t(33) = 4.1752 ***P = 0.00020	t(42) = 3.4225 **P = 0.0014	t(37) = -1.0700 P = 0.2915	t(30) = 1.1090 P = 0.2763
	Reduction	t(33) = 1.6243 P = 0.11384	t(42) = 0.3161 P = 0.7535	t(37) = -1.1435 P = 0.2602	t(30) = 0.6566 P = 0.5165
	Spike number (/500msec)	t(33) = 1.2298 P = 0.22746	t(42) = -0.7413 P = 0.4627	t(37) = 0.4097 P = 0.6844	t(30) = 0.0554 P = 0.9562

Fast-spiking interneuron		Visual cortex L2/3 fast-spiking interneuron		Hippocampal CA1 fast-spiking interneuron	
		P7-8	P18-22	P7-8	P16-18
	Input resistance	t(17) = 0.8747 P = 0.3939	t(22) = -0.5791 P = 0.5684	t(26) = 0.0474 P = 0.9626	t(17) = 1.1128 P = 0.2813
	Resting membrane potential	t(17) = -0.5032 P = 0.6213	t(22) = -0.2529 P = 0.8027	t(26) = 0.2843 P = 0.7784	t(17) = -0.9869 P = 0.3376
	Injected currents	t(17) = -1.3552 P = 0.1942	t(22) = 0.7787 P = 0.4445	t(26) = -0.1722 P = 0.8647	t(17) = -0.3914 P = 0.7004
Single-APs	Threshold	t(17) = 0.3485 P = 0.7318	t(22) = -3.1077 **P = 0.0051	t(26) = 2.8850 **P = 0.0078	t(17) = 0.7377 P = 0.4708
	Peak amplitude	t(17) = 0.8793 P = 0.3915	t(22) = 2.0676 P = 0.0506	t(25) = 1.2701 P = 0.2158	t(17) = -0.9424 P = 0.3592
	Afterhyperpolarization	t(17) = -0.1870 P = 0.8539	t(22) = -0.1888 P = 0.8520	t(26) = -0.6785 P = 0.5035	t(16) = 1.9948 P = 0.0634
	Half-width	t(17) = 0.0435 P = 0.9658	t(22) = -0.8992 P = 0.3783	t(26) = -0.6843 P = 0.4998	t(17) = -0.4230 P = 0.6776
Train-APs	1st spike peak amplitude	t(16) = 0.7438 P = 0.4678	t(22) = 1.7425 P = 0.0954	t(26) = 1.3365 P = 0.1930	t(17) = -0.9214 P = 0.3697
	1st spike width	t(17) = -0.1442 P = 0.8871	t(22) = -1.1528 P = 0.2614	t(26) = -0.5195 P = 0.6078	t(17) = -0.6796 P = 0.5059
	2nd spike peak amplitude	t(17) = 0.3672 P = 0.7180	t(22) = 1.4735 P = 0.1548	t(26) = 1.3529 P = 0.1877	t(17) = 0.1795 P = 0.8597
	2nd spike width	t(17) = -0.4430 P = 0.6634	t(22) = -1.4794 P = 0.1532	t(26) = -0.6357 P = 0.5305	t(17) = -0.3884 P = 0.7025
	Last spike amplitude	t(17) = 1.5743 P = 0.1338	t(22) = 0.2951 P = 0.7707	t(26) = 0.3426 P = 0.7346	t(17) = -0.3914 P = 0.7004
	Last spike width	t(17) = -0.1796 P = 0.8596	t(22) = -1.4216 P = 0.1692	t(25) = 0.7712 P = 0.4478	t(17) = 0.5249 P = 0.6065
	Reduction	t(17) = 0.4652 P = 0.6477	t(22) = -1.3039 P = 0.2058	t(26) = 0.0645 P = 0.9491	t(17) = 1.7026 P = 0.1069
	Spike number (/500msec)	t(17) = -0.5675 P = 0.5778	t(22) = 1.1695 P = 0.2547	t(26) = -1.5172 P = 0.1413	t(17) = -0.3834 P = 0.7062
